# The Spectrum of Cognitive Impairment in Atypical Parkinsonism Syndromes: A Comprehensive Review of Current Understanding and Research

**DOI:** 10.3390/diseases13020039

**Published:** 2025-01-31

**Authors:** Kurt A. Jellinger

**Affiliations:** Institute of Clinical Neurobiology, Alberichgasse 5/13, A-1150 Vienna, Austria; kurt.jellinger@univie.ac.at; Tel./Fax: +43-1-5266534

**Keywords:** atypical parkinsonism, multiple system atrophy, corticobasal degeneration, progressive supranuclear palsy, cognitive impairment, brain network disruptions, pathogenic questions

## Abstract

Multiple system atrophy (MSA), progressive supranuclear palsy (PSP), and corticobasal degeneration (CBD) are the most common atypical parkinsonism (AP) syndromes. They are clinically characterized by varying combinations of levodopa-poorly responsive parkinsonism, motor, cerebellar, and other signs. They are associated with a wide spectrum of non-motor symptoms, including prominent cognitive impairment such as global cognitive deficits, memory, executive, attentional, visuospatial, language, and non-verbal reasoning dysfunctions. Within the APs, their cognitive functioning is distributed along a continuum from MSA with the least impaired cognitive profile (similar to Parkinson’s disease) to PSP and CBD with the greatest decline in global cognitive and executive domains. Although their pathological hallmarks are different—MSA α-synucleinopathy, CBD, and PSP 4-repeat tauopathies—cognitive dysfunctions in APs show both overlaps and dissimilarities. They are often preceding and anticipate motor dysfunctions, finally contributing to reduced quality of life of patients and caregivers. The present paper will review the current evidence of the prevalence and type of cognitive impairment in these AP syndromes, their neuroimaging, pathogenic backgrounds, and current management options based on extensive literature research. Cognitive dysfunctions in APs are due to disruption of prefronto-subcortical and striato-thalamo-cortical circuitries and multiple essential brain networks. This supports the concept that they are brain network disorders due to complex pathogenic mechanisms related to the basic proteinopathies that are still poorly understood. Therefore, the pathophysiology and pathogenesis of cognitive impairment in APs deserve further elucidation as a basis for early diagnosis and adequate treatment of these debilitating comorbidities.

## 1. Introduction

Atypical parkinsonism (AP) syndromes are heterogeneous neurodegenerative disorders that differ from Parkinson’s disease (PD) mainly because of their clinical and pathological features as well as genetic variabilities [1,2,3].

Among a wide variety of APs, multiple system atrophy (MSA), progressive supranuclear palsy (PSP), and corticobasal degeneration (CBD) are the most common ones [4,5]. These are relatively rare disorders clinically characterized by levodopa-poorly responsive progressive parkinsonism and varying combinations of motor and non-motor symptoms, including autonomic and cerebellar symptoms associated with cognitive, behavioral, and other neuropsychiatric disturbances [3,6,7,8]. Many of these symptoms may be present not only in the early phase of the disease but also frequently anticipate motor symptoms and do not appear to be related to the severity of motor dysfunctions. Since cognitive dysfunctions are among the main factors that determine a low quality of life for both patients and caregivers, they should always be assessed as early as possible. Despite their different pathological background (MSA—α-synucleinopathy, PSP and CBD—4-repeat (4R) tauopathies) their cognitive functioning shows a continuum from the least impaired MSA to PSP and CBD, although there are similarities and overlaps. Therefore, it is unclear whether each type of AP displays specific cognitive symptoms or whether they develop secondary to the severity/progression of the basic disorder or as non-specific comorbidities. Their pathogenesis is still poorly understood. In view of these open questions, the present paper is intended to review the available data on the prevalence and type of neuropsychiatric disturbances in patients with MSA, PSP, and CBD, their structural and functional neuroimaging, pathogenic mechanisms, and current and future management options, based on extensive research of the relevant literature.

## 2. Methods

A systematic review of cognitive disturbances in MSA, PSP, and CBD was performed using PubMed, Google Scholar, and the Cochran Library to identify all papers published on this topic between 1990 and November 2024. Since this is not a systematic but a narrative review, the PRISMA statements for reporting systematic reviews were not followed. Search was performed according to the following MeSH keywords: MSA, PSP, CBD, cognition, cognitive impairment, memory, attention-executive dysfunctions, planning, decision-making, visuospatial abilities, language, non-verbal reasoning, processing speed, structural and functional neuroimaging, pathogenic factors. Only articles in English or with English summaries in peer-reviewed journals were considered, and full articles were examined to consider their relevance based on the inclusion criteria. Exclusion criteria: case reports, conference abstracts and reports, commentaries, letters, duplicate studies, non-English articles without English abstracts, and studies focusing on neuropsychiatric disorders and symptoms, including psychoses, hallucinations, delusions, and other severe neuropsychiatric disorders. Not considered were mild cognitive impairment in MSA and depression in MSA, PSP, and CBD, which have been reviewed recently [9,10,11,12]. Furthermore, cognitive impairment in PD was not considered, since it also has been reviewed recently [13,14]. The reference lists of the papers were also examined. All citations of the studies identified by the search were inspected and grouped according to the specific topics.

## 3. Results

### 3.1. Cognitive Impairment in Multiple System Atrophy

Unlike other synucleinopathies, MSA has previously not been associated with significant cognitive impairment (CI), which has even been considered an exclusion criterion for its diagnosis [15]. However, a position statement of the Movement Disorder Society (MDS) stated that CI is a recognized feature in MSA, occurring in 17–47% of MSA patients, while severe dementia is rare [16]. Since CI has been underestimated in MSA, not all patients have undergone formal cognitive assessment, and, therefore, the frequency could be higher than reported in several studies.

*Prevalence of CI in MSA*. While about 50% of MSA patients exhibit distinct types and levels of CI [17], they account for 35% to 37% of pathologically confirmed MSA [18,19,20,21]. The degree of CI ranges from mild to severe decline and affects executive, attentional, visuospatial, and verbal functions, while memory is less often and less severely impaired, affecting 11% to 16% [21,22], but only 7% (95%-CI 0–12%) of pathologically confirmed MSA [21]. CI may occur in early stages but is generally common in advanced disease [23] and is often correlated with disease duration [24]. While in a recent clinical study, executive functions (EFs) and verbal memory were primarily affected [25], according to others, attention was the main altered cognitive domain [26]. Patients with the parkinsonism variant of MSA (MSA-P) displayed higher deficits in EFs (i.e., cognitive rigidity) than healthy controls (HCs) [27,28,29,30].

*Cognitive domains affected in MCI*. Comparative cognitive studies in MSA-P and the cerebellar variant of MSA (MSA-C) suggested that both groups have, at least in part, different cognitive profiles. While several studies could not find significant differences in the cognitive performance between groups [30,31,32], others revealed more severe and widespread cognitive dysfunction in MSA-P patients [33,34], probably due to prefrontal impairment [35]. Both groups present impaired executive and visuospatial functions, while the attention deficit is predominant in MSA-C [36]; the latter also obtained lower scores for EFs and verbal memory [32]. In MSA-P, executive and attentional functions, episodic memory, language, execution, visuospatial abilities, and temporo-spatial orientation were compromised, while MSA-C dysfunction was restricted to attentional and executive domains [34].

Others reported more severe impairment in mental speed, working memory, EFs, focused attention, and repetition abilities in MSA-P patients, while MSA-C patients had more deficits in new learning, immediate recall, verbal fluency, sustained attention, spatial planning, and psychomotor speed [28,37]. Execution dysfunction was more pronounced among MSA-P APOE ε4 carriers [26].

*Risk factors for CI in MSA*. Orthostatic hypotension, but not its severity, increases the risk of CI in MSA (and PD) but is not associated with cerebrovascular pathology [38], while cerebral small vessel disease may be correlated with CI [39] and multiple vascular risk factors have a cumulative impact on CI in MSA [40]. However, the majority of MSA patients (62%) have no significant co-morbid pathologies, which may be unique among neurodegenerative diseases [41]. Whereas neuronal cortical inclusion burden in hippocampus, parahippocampus, and perirhinal regions are associated with memory impairment [42,43,44], Alzheimer disease (AD)-related neuropathological changes (ADNC), cerebral amyloid angiopathy (CAA), and other cerebrovascular lesions did not differ between MSA cases with and without CI [45]. However, some patients suffering MSA with longer disease duration show p-tau deposits mainly in hippocampal CA2/3 pyramidal neurons, suggesting that α-synuclein (αSyn) expression may influence tau phosphorylation in MSA [46].

ADNC has been reported in only 2 out of 35 (7%) of autopsy-proven cases of MSA [18], whereas in two cases of combined MSA and AD, a few neurons shared αSyn and tau deposition [47]. Among 50 autopsy-proven MSA cases (33 MSA-P, 15 MSA-C) with a mean age at death of 60.5 ± 7.8 years, moderate CI in 35% and severe dementia in a single case, moderate cortical tau pathology (Braak II-III) was seen in 30%, PART and AD (Braak V) in one case each. Additional Lewy pathology (8.4%) was higher than in the control group [19].

### 3.2. Cognitive Dysfunction in Progressive Supranuclear Palsy

CI is a common feature in PSP [23,48], a 4-repeat tauopathy, the clinical criteria [49,50] of which have been expanded by several phenotypes [51] based on different pathologies [52,53]. Attention-dysexecutive symptoms may be among the earliest presenting evidence of this disorder [54], starting more than three and up to 10 years before the diagnosis of PSP. These patients present a broad variety of motor and non-motor/cognitive, behavioral, and mood symptoms years before diagnosis [55,56]. The recent criteria for PSP [49] do not list dementia among the supporting diagnostic features, although its presence may be relevant to considering this diagnosis together with other supporting features [57]. CI is evident in its early stages in about 50%, with executive dysfunction in 40% [23]. The pattern of early cognitive changes in PSP is typically a dysexecutive frontal syndrome with slowness of cognitive processes [48,58]. This and verbal fluency changes are prominent, but other cognitive functions, like memory, naming, construction, visuospatial abilities, and social cognition, can also be affected [48,59]. Verbal fluency is among the most frequently impaired EFs in PSP and is strongly associated with midbrain atrophy [60]. Language deficits such as progressive non-fluent aphasia may also be the first presentation or can appear later in the course of the disease, with speech-language disorders being constant symptoms [61].

Impaired cognitive profiles vary among PSP phenotypes. The Richardson’s syndrome/classic PSP (PSP-RS) group is affected by greater impairment of EFs and processing speed compared to the subcortical phenotypes (PSP with predominant frontal/cognitive presentation/PSP-F, PSP with predominant parkinsonism/PSP-P, and PSP with predominant gait freezing and motor blocks/PSP-PGF) [62]. 81% showed executive dysfunction, 64% cognitive slowing, 55% ineffective memory, 33% impaired word finding, 22% disorientation, and 17% visuospatial impairment [63]. Other frequently affected functions are visuospatial perception, attention, and construction [64]. In a cohort of autopsy-confirmed cases of PSP, CI was reported in 74%, most frequent in PSP-F (100%), followed by PSP-RS (75%), PSP-P, and PSP-CBS (PSP with predominant corticobasal syndrome) (57% each) and 80% in unclassified cases [65]. A review reported CI in 32% of all clinical phenotypes, ranging from 0% in PSP-P to 83.3% in PSP-CBS, 47.8% in PSP-RS, 66.7% in PSP-F, and 28.6% in PSP with predominant oculomotor dysfunction (PSP-OM) [66]. Furthermore, there is a broad deficit in social cognition, impairing recognition of emotion and mind [67]. Frontal impairment was observed in 60% and dementia in 20%. The 10-year cumulative probability of developing dementia was 71% in PSP (54% in PD) [6]. A recent systematic review found that PSP patients had lower scores on episodic memory compared to HCs and MSA ones, with only little differences from PD but much less than in typical AD [68]. Executive tests identified deficits in reasoning, planning, set shifting, verbal fluency, information processing speed, and response initiation, associated with frontal lobe dysfunction [58]. Executive, language, and visuospatial abilities decline longitudinally in PSP but not in MSA and PD [69].

### 3.3. Cognitive Impairment in Corticobasal Syndrome (CBS)/Corticobasal Degeneration (CBD)

While motor symptoms of CBD are well recognized, the fact that most, if not all, patients develop CI has not been generally accepted by some [70], although it has been included in the diagnostic criteria of CBD [71], a 4-R tauopathy for which the term “CBD” is limited to the underlying neuropathological disorders, while “CBS” refers to the clinical syndrome that shows a variety of phenotypes [72]. CI (and other neuropsychiatric symptoms) are common clinical features of CBS [73,74]. Cognitive deficiencies are frequent initial symptoms and may appear up to 8 years prior to motor symptoms, depending on the phenotypic variant [75,76]. The median intervals from the initial symptoms to the onset of key milestones have been described as follows: gait disturbances 0.0 years, behavioral changes 1.0 year, falls 2.0 years, CI 2.0 years, speech impairments 2.5 years, supranuclear gaze palsy 3.0 years, urinary incontinence 3.0 years, and dysphagia 5.0 years; the median survival was 7.0 years. Only 50% were diagnosed as CBD/CBS at the final presentation, while pathology detected CBD (33.3%), PSP (29.2%), and AD (12.5%). In the common course, CBD/CBS CI manifested after 3 years, while AD-CBS patients showed it already 1 year after disease onset [77].

Characteristic CI in CBS involves the majority of cognitive domains, including attention, EFs, processing speed, and working memory, with relative preservation of verbal and non-verbal memory [78], while a subset of CBD patients shows a cognitively predominant syndrome, with executive and visuospatial dysfunctions and apathy, that can be mistaken for AD or frontotemporal lobe degeneration (FTLD) [79]. More than 80% of CBD patients present executive dysfunctions [77].

Dynamic aphasia with reduced voluntary speech is an early sign of CBD [80]. Aphasia is typically non-fluent, while semantic memory appears relatively preserved [81]. A specific pattern associated with extrapyramidal motor abnormalities is persistent impairment of executive functioning, progressive worsening of language performance, and moderately preserved memory [82]. Other symptoms of CBD include apraxia, alien hand syndrome, apathy, and frontal lobe dysexecutive changes with disinhibition [83]. Constructional and visuospatial difficulties, acalculia and frontal dysfunction, and apraxia of speech, together with motor speech disorders (verbal fluency), are frequent symptoms [84,85]. Apraxia of speech is characterized by slow speech rate, abnormal prosody, distorted sound segmentations, repetitions, and syllable segmentations [86]. In a large cohort of probable CBS, aphasia was the second most probable impairment, present in 67.7%, following apraxia (96.8%), associated with impaired verbal fluency [87]. These patients, in addition to having visuospatial deficits and alien hand syndrome, show severe language disorder, characterized by non-fluent speech, speech apraxia with prominent stuttering-like dysfluencies, agraphia, lack of word-finding, and defective sentence repetition. A recent clinico-pathological study compared classic-onset and speech/language-onset CBS: The classic-onset form presents with asymmetric limb apraxia (alien limb phenomenon), dystonia, apraxia, and myoclonus, while the speech/language-onset CBS has a higher frequency of vertical supranuclear gaze palsy. Pathological lesion burden (including astrocytic plaques) does not differ between the two clinical subtypes [88]. In an FDG-PET study, the CBS AD group demonstrated worse cognitive performance, mostly concerning attention, memory, and visuospatial domains, and displayed more myoclonus and hallucinations; the CBS non-AD group presented more often with limb dystonia and motor dysfunction [89]. Aphasia may also present a ’mixed’ primary progressive aphasia (PPA), a combination of nonfluent and logopenic variants, associated with apraxia of speech, stuttering, and agraphia [90].

### 3.4. Differences and Overlaps of CI in Atypical Parkinsonian Syndromes (Table 1)

CI is a common clinical feature in all three APs, observed in 31.8% of patients with MSA, in 62% in PSP, and in 90% in CBD [77]. Others reported 35–37% CI in pathologically confirmed MSA [20,21] and between 30% and 83% for PSP (depending on the phenotype) [66]. Dementia was observed in 20% of the MSA group and in approximately 57% of PSP, with impairment of a single and multiple domains in 40% each, while in MSA the figures were 28.6% and 13.5%, respectively. Although with regard to global CI and executive dysfunction PSP performed worst among the three groups, their cognitive profiles were similar, with the main impairment of initiation and perseveration. This indicates a high level of CI in PSP but also points to comparable dysfunction in a substantial part of MSA patients [23]. This was confirmed by later studies, showing that PSP patients have more severe CI compared to MSA patients [91].

**Table 1:** Major cognitive disturbances in atypical parkinsonian disorders.

Impaired Function	MSA	PSP	CBD	
Episodic memory	++/+	++	+	MSA-P > MSA-C; PSP > MSA
Working memory	+	++	++	MSA-P > MSA-C; CBD > PSP
Semantic memory	+	+/–	+	
Verbal memory	+/++	+	+	MSA-P > MSA-C
Executive function	++	++	++	CBD > MSA; MSA-P > MSA-C
Attention	++	++	++	MSA-C > MSA-P
Immediate recall	+/++	+	+	MSA-C > MSA-P
Mental/psychomotor speed	++/+	++	+	MSA-P > MSA-C; PSP > CBD
New learning	+	+	+	MSA-P > MSA-C
Repetition abilities	++/+	+	++	MSA-P > MSA-C
Aphasia, non-fluent	–	++	++	CBD > PSP
Speech apraxia	–	+	++	CBD > PSP
Agraphia	– –	+	++	
Logopenia	–	+	+	
Dysarthria	–	+	++	CBD > MSA
Verbal fluency	+	++	++	CBD > PSP > MSA
Semantic fluency	+	+	+	
Processing speed	+ –	+	+	
Constructional ability	+	++	+	
Spontaneous verbal recall	+	+	+	
Decision-making	+	+	+	
Speech apraxia	–	?	++	
Semantics	+	+	+	
Acalculia	–	+	+	
Temporo-spatial orientation	+/++	++	+	MSA-P > MSA-C
Visuospatial skills	+/++	++	+/++	PSP > MSA; PSP > CBD; MSA-P > MSA-C
Verbal retrieval	+/++	++	+	MSA-P > MSA-C
Language performance	?	+	+	
Spatial orientation	+	+	+	MSA-P > MSA-C
Social cognition	+	+	+	
Naming	+	+	+	
Spatial planning	+	+	+	MSA-C > MSA-P
Perseveration	+	+	+	
Inhibition	?	+	+	

MSA: multiple system atrophy; MSA-P: MSA parkinsonism variant; MSA-C: MSA cerebellar variant; PSP: progressive supranuclear palsy; CBD: corticobasal degeneration.

Cognitive deficits are frequent initial symptoms in APs (22% in MSA and 50% in PSP), attention-executive dysfunctions and apathy being among the earliest evidence of CI in both MSA and PSP [54,55], while reduced voluntary speech is one of the early signs of CBD [80]. In MSA, CI affects primarily EFs, attention, and visuospatial and verbal functions/memory, while in PSP, executive dysfunction, visuospatial abilities, memory, orientation, word finding, and processing speed are predominantly involved, although the impaired cognitive profiles vary considerably among PSP phenotypes [62]. The same problem arises in CBD, where disorders of executive/attention, processing speed, and working memory are typical CIs of classical CBD, whereas for other subtypes (non)fluent aphasia, speech apraxia, and other language disorders are predominant. A prospective longitudinal clinicopathological study analyzing differences between CBD and PSP showed that motor speech impairment was the most prominent common feature, while a trend towards greater sentence deficits was found in the nonfluent/agrammatic variant of primary progressive aphasia (nfvPPA)-CBD group. Motor speech impairment was the most prominent common feature for patients with nfvPPA underlying PSP and CBD pathology, while greater working memory deficits were seen in CBD patients [92]. In conclusion, cognitive dysfunctions occur in MSA, PSP, and CBD, showing frequent overlap between the three disorders [93]. However, they differ in the frequency and degree of the affected cognitive domains, which obviously reflects the pattern and intensity of the underlying pathologies, such as a higher degree of cortical involvement in both PSP and CBD that, however, show distinct clinical and pathological profiles [94,95]. In the CBD group, due to its variable phenotypic presentation, comparison may be difficult, although the subset of CBD-Cog (cognitive rather than motor predominant features) shows more severe cognitive than motor features with predominant executive and visuospatial dysfunctions that can mimic FTLD or AD, although their language features do not differ from classical CBD-CBS [79].

### 3.5. Neuroimaging Findings

#### 3.5.1. Structural Changes (Table 2)

The diagnostic accuracy of the International Parkinson and MDS MSA criteria is increased by MRI markers [96]. MRI studies display progressive atrophy of the cerebral cortex and striatum [97], and there is a significant correlation between various cognitive domains and atrophy of frontotemporal cortical areas, insula, caudate, thalamus, and cerebellum [37]. These data are in accordance with previous studies that suggested that lesions in the frontal lobe play an essential role in CI in MSA [31,98,99] and that frontal dysfunction driven by fronto-striatal degeneration may lead to CI in MSA [100]. The dorsolateral prefrontal cortex is closely related to EFs [101]; together with attention and language processing, it is positively related to volume reduction in the dorsolateral prefrontal lobe, causing striato-frontal dysfunction [102]. Widespread frontostriatal white matter (WM) reduction in fractional anisotropy in MSA-P seems to contribute to executive dysfunction in MSA-P [27]. MSA-CI patients exhibited cerebellar atrophy, decreased fractional anisotropy, and decreased diffusivity in both the cerebrum and cerebellum, with a significant decrease in the anterior corpus callosum [103].

**Table 2:** Neuroimaging findings associated with CI in APs: structural MRI.

Findings	References
**MSA**	
Atrophy, fronto-temporo-parietal cortex, insula, caudate nucl., thalamus, and cerebellum	[37]
Atrophy, dorsolateral prefrontal cortex	[101,102]
Atrophy, left temporal lobe (inferior temporal gyrus, superior temporal gyrus)	[104,105,106]
Atrophy, hippocampus (sector CA2/3)	[107]
Reduction, frontostriatal WM	[27]
WM damage (anterior corpus callosum), cerebellar atrophy	[103]
Reduced WM density in cerebellum and brainstem	[108]
**PSP**	
Atrophy, frontotemporal cortex, hippocampus, basal ganglia, midbrain	[109,110,111]
Atrophy, prefrontal cortex, temporal pole	[99]
Atrophy, frontal, precentral cortex, insula, superior cerebellar peduncle	[112]
Atrophy, frontal lobe	[113]
Atrophy, medial frontal cortex, cingulum, insula, striatum, thalamus, and midbrain	[67]
Atrophy, midbrain	[94]
Atrophy, WM (corpus callosum, longitudinal fascicles)	[114,115]
WM lesions (corpus callosum, superior cerebellar peduncle, corona radiata)	[116]
Widespread macro- and microscopic WM changes	[117]
**CBD**	
Asymmetric atrophy, frontotemporal cortex, midbrain	[118]
Asymmetric dorsal premotor and perirolandic cortex, midbrain	[119]
Asymmetric prefrontal, fronto-temporal, parietal, and cingulate cortex	[75,120]
Asymmetric frontal lobe, operculum	[80]
Asymmetric prefrontal cortex, frontal WM	[92]
Asymmetric superior parietal lobule and left angular gyrus	[121]
Atrophy, left superior temporal, perisylvian cortex	[122]
Atrophy, frontal and temporal cortex and midbrain	[123]
Atrophy, nucleus basalis of Meynert	[124,125]
Lesion, left WM (frontal operculum)	[80]
Widespread macro- and microscopic WM changes	[117]
WM reduction, insular putamen, thalamus	[126]

WM: white matter.

The left temporal lobe is involved in many cognitive processes, including visual processing and delayed memory (inferior temporal gyrus) [104,127], language processing, and semantic comprehension (superior temporal gyrus) [105,106,128]. Recent studies indicated that there is an association between CI and hippocampal subfield volumes in MSA. The hippocampal volume in the CA2/3 subfield decreased in patients with MSA-CI even at early disease stages, and the right CA2/3 volume correlates significantly with the Frontal Assessment Battery scores [107].

Other studies suggested a role for the putamen and cerebellum in the cognitive changes in MSA, probably due to their connections with the cortex that are disturbed due to reduced WM density in the cerebellum/brainstem [108]. Whole-brain deep and superficial WM diffusivity abnormalities mirror the widespread aggregation of αSyn in oligodendrocytes and are associated with CI and other clinical symptoms [129]. MSA-CI exhibits significantly more WM damage than cognitively preserved and PD ones [130].

Brain imaging in PSP shows atrophy of the midbrain that is a common feature of all PSP groups [94] and is associated with verbal fluency [60]. Patients with CI show reduced gray matter (GM) volumes in various cortical and subcortical regions, including frontal, temporal, and hippocampal areas; basal ganglia; midbrain; and cerebellum, with reduced cortical thickness in the left entorhinal and fusiform gyrus [109]. The earliest atrophy affects the brainstem and subcortical regions, progressing into the superior cerebellar peduncle and deep cerebellar nuclei, and rostral to the cortex, where it progresses to the insula, the frontal lobe, before spreading to the temporal, parietal, and finally the occipital lobe [112]. This in vivo model is in accordance with the postmortem staging of tau pathology in PSP [131]. PSP-CI is associated with GM atrophy in frontotemporal regions, the thalamus, and the globus pallidus, with predominant involvement of the midbrain, basal ganglia, and cerebellum [110]. Executive dysfunction is related to bilateral atrophy of the prefrontal/precentral cortex, temporal poles, and dorsolateral anterior cortex [132] and significant frontal atrophy [113]. Cognitive-behavior deficits are linked to synapse loss in the superficial and granular layers of the inferior frontal cortex [133]. Damage to the limbic and paralimbic systems correlated with memory impairment [134]; the frontal operculum, insula, pre- and postcentral gyri, superior frontal gyri, and anterior parietal lobule are also affected [135].

PSP clinical variants show various patterns of involvement of cortical and subcortical areas, suggesting different patterns of tau pathology spreading through the brain. PSP-cortical phenotypes (PSP-PL, PSP-CBD, and PSP-F) show volume loss in frontal lobes, PSP-F (and behavioral variant frontotemporal dementia) show similar changes with atrophy in frontal medial cortex, cingulum, insula, limbic structures, and anterior temporal lobe) while in subcortical phenotypes (PSP-P, PSP-OM), lesions predominantly involve striatum, thalamus, substantia nigra (SN), globus pallidus (GP), and subthalamic nucleus (STN). PSP-PL shows predominant volume loss and tau burden in the motor cortex and supplementary motor area, while PSP-P shows greater involvement of the putamen and SN [67].

WM atrophy involves the corpus callosum, dorsomedial midbrain, and periventricular WM [115]. Diffusion tensor MRI reveals damage to the corpus callosum, cingulum, bilateral uncinate fascicles, and inferior longitudinal fascicle, related to executive dysfunction [114]. Lesions involving the corpus callosum, corticospinal tracts, superior cerebellar peduncle, fronto-occipital, superior longitudinal, and uncinate fascicles bilaterally are associated with cognitive deficits [132], as are increased mean diffusivity in the superior cerebellar peduncle, anterior, superior, and posterior corona radiata, and the body of the corpus callosum [116]. Fixel-based analysis detects fiber-specific micro- and macroscopic WM alterations in both PSP and CBD, monitoring the progressive tau-related WM changes in vivo and indicating strong correlations with cognitive (and motor) dysfunctions in both diseases [117].

In CBD, structural neuroimaging often displays asymmetric cortical and subcortical abnormalities that underlie the pathophysiology of asymmetric symptoms, in particular, midbrain and frontotemporal atrophy [118]. Early cognitive (and behavioral) dysfunctions are associated with asymmetric atrophy of the dorsal premotor and perirolandic cortex and striatum [75,119], recent memory impairment, and right hemiparkinsonism with WM volume reduction in the frontal lobe between the left supplementary motor area and the frontal operculum [80]. CBS has higher GM volume loss in the cortex and basal ganglia and synapse loss that is more severe and widespread in the group with negative β-amyloid deposition. Asymmetry of synapse loss is in line with the clinically most affected side [136].

Dynamic aphasia with reduction in voluntary speech, an early symptom of CBD, is related to left-dominant WM reduction in the frontal lobe between the supplementary motor area and frontal operculum [80]; verbal fluency impairment is related to atrophy of the left perisylvian cortex, and visuospatial dysfunction is related to that of the left posterior superior temporal gyrus [137]. Speech apraxia is associated with atrophy of the premotor and supplementary motor areas [122], motor speech and memory impairment with atrophy of the prefrontal cortex and frontal WM [92]. Apraxia is related to widespread bilateral clusters of atrophy, mainly in superior parietal lobes and the left angular and supramarginal gyri [138].

Structural damage to the nucleus basalis Meynert is associated with CI in CBD (as well as in PD) and may represent a promising noninvasive in vivo marker of cognitive decline in these disorders due to the involvement of the cortical cholinergic innervation [124,125]. CBS/PSP-AD demonstrates atrophy in AD signature areas and the brainstem, while CBS/PSP-no AD patients display atrophy in frontal and temporal areas, GP, and the brainstem compared to HCs [123]. The high variety of structural brain changes among the clinical phenotypes of CBD has been discussed recently [93].

#### 3.5.2. Functional/Brain Network Changes

Network connectivity alterations arise as early as the presymptomatic phase in MAPT-associated clinical syndromes [139]. Functional MRI studies suggested that CI in MSA may be related to dysfunction in fronto-striatal connections [100] and to the dorsolateral prefrontal default mode network (DMN) and disruption of the dorsolateral prefrontal-insular network [102] (Table 3). Connections between the right dentate nucleus and cerebellum [140], from putamen and cerebellum to the cortex [108], and functional connectivity (FC) of the cerebello-prefrontal and cerebello-amygdala networks are also interrupted [17,141]. A recent study suggested that altered spontaneous activity in the dorsolateral prefrontal cortex (DLPFC) and the lower one in the cerebellum, as well as corresponding disruption in the DLPFC to the inferior parietal lobe and of cerebello-cerebral connections, may be simultaneously involved in early cognitive decline in MSA [142]. This corresponds to the idea that CI in MSA predominantly is related to disorders in the frontal executive domain [23]. The prefrontal lobe, specifically the DLPFC, was suggested to be related to EFs [101], since it shows volume reduction [100], hypoperfusion [35], and other abnormalities. MSA-P patients show decreased FC between DLPFC and cerebellar dentate nucleus, a disordered striato-thalamocortical network, and enhanced FC between the dentate nucleus and posterior cingulate cortex, which is associated with executive and emotional processes [143]. A meta-analysis of αSyn patients (including MSA) demonstrated imbalanced FC among subcortical networks, the cerebellum, and fronto-parietal networks that involve executive control (and motor functioning); association with hypoconnectivity in DMN and the ventral attention network was involved in cognition and attention [144].

Both MRI and PET patterns may predict global cognition in PSP [145] (Table 4). fMRI displays altered FC between the brainstem and cerebellum, diencephalon and basal ganglia, and connections, particularly between and within the cortico-subcortical and cortico-brainstem networks. PSP patients show reduced mean midbrain-cortical FC and widespread disruption of cortico-subcortical connectivity [146]. A systematic review showed significant reduction in both cortical and subcortical regions, including frontal and superior temporal areas, insula, striatum, midbrain, and cerebellum, causing disruption of cortico-subcortical circuits, in PSP causing involvement of frontal-subcortical circuits [111]. Cognitive deficits and apathy in PSP are related to dysfunction of the prefronto-subcortical circuits (i.e., dorsolateral prefrontal and limbic circuits) [147]. Pathological involvement of cortico-basal ganglia-thalamocortical loops and thalamus dysfunction contribute to behavioral and cognitive disturbances [148]. PSP patients show widespread axonal loss in the superior cerebellar peduncles, dentato-rubro-thalamic tracts, thalami, and frontal WM [149].

**Table 4:** Neuroimaging findings associated with CI in PSP: functional MRI.

Findings	References
Reduced FC, brainstem —cerebellum	[146]
Reduced FC, diencephalon—basal ganglia	
Reduced FC, cortico-subcortical network	
Reduced FC, cortico-brainstem network	
Reduced FC, midbrain-cortical network	
Disruption, cortico-subcortical network	[111]
Disruption, fronto-subcortical network	
Dysfunction, prefronto-subcortical network (DLPFC-limbic circuit)	[147]
Reduced FC, cortico-basal ganglia-thalamo-cortical loop	[148]
Disruption, dentato-rubro-thalamic tract	[149]
Reduced FC, prefrontal-paralimbic circuit	[150]
Reduced FC, cortex-subcortical tau epicenters	[151]

CI: cognitive impairment; PSP: progressive supranuclear palsy; FC: functional connectivity; DLPFC: dorsolateral prefrontal cortex.

Disrupted structural connectivity of frontal deep GM pathways is characteristic of PSP [152] and correlates with disorders of cognitive performance [153]. Advancing functional disconnectivity in subcortical-anterior cortex is followed by later-stage subcortical-posterior cortical changes with a decline of prefrontal-paralimbic FC [150]. Combined resting-state fMRI assessing connectivity with 4R-tau deposition patterns revealed inter-regional FC associated with higher inter-regional correlations of both tau-PET and postmortem tau patterns, indicating their association with the connectivity of subcortical tau epicenters [151]. In PSP-RS, disorders of the structural connectivity of the whole-brain connectome are present, in particular affecting frontal and deep GM connections between basal ganglia and between the frontal lobe and basal ganglia, supporting the concept that PSP is a brain network-based disorder.

In CBD, multiple neuronal networks related to movement and execution are functionally involved [154] (Table 5). Bilateral involvement in the dorsal attentional network (DAN) causes attentional neglect [155]. FC between the DAN and frontoparietal network is reduced, while there is increased connectivity between the DMN, DAN, and motor networks and decreased FC within these networks [156]. Interconnectivity is reduced in the right central operculum, middle temporal gyrus, and posterior insula, but increased in the anterior cingulum, medial superior frontal gyrus, and bilateral caudate nuclei. Multimodal MRI studies detecting diffuse WM volume reduction in the insula, thalamus, and putamen suggest that both structural and connectivity abnormalities are important [126], and combined fMRI connectomics associated with tau-PET revealed inter-regional FC associated with higher inter-regional correlations between functional deficits and tau levels, indicating that specific tau patterns correlate with the connectivity of related epicenters [151]. The DMN shows the greatest disconnection in CBS/PSP-AD compared to CBS/PSP-no AD and HCs, while the thalamic network is most affected in CBS/PSP-no AD [123]. Connectivity between deep nuclei, motor regions, and the thalamus is the most important for APs (and PD) [157].

#### 3.5.3. Metabolic and Neuromodulatory Changes

MSA, besides putaminal and cerebellar atrophy, demonstrates nigrostriatal and olivocerebellar hypometabolism related to αSyn cytoplasmic inclusion (GCI) burden; PSP typically shows midbrain atrophy on structural imaging, whereas PET depicts frontal lobe hypometabolism and confirms underlying tauopathy; CBD displays asymmetric atrophy of the superior parietal lobule and corpus callosum, whereas FDG- and tau-PET demonstrate asymmetric hypometabolism and subcortical involvement contralateral to the side of clinical deficits [158]. FDG-PET provides added value for detection of PSP in patients with inconclusive MRI, regardless of PSP phenotype [159].

MSA-P shows reduced metabolism in the bilateral cortex and alterations in corticostriatal WM integrity associated with executive dysfunction [27]. MSA-CI patients exhibit hypometabolism in the left middle and superior frontal lobes [160], but hypometabolism of the limbic regions is also associated with CI in MSA [161].

Reduced scores for attention, EFs, and language correlate positively with the metabolism in the superior-inferior frontal gyrus and the cerebellum, but negatively with that in the insula and fusiform gyrus (*p* < 0.001), while no significant differences in neuropsychological performance and frontal metabolism are found between patients with MSA-P and MSA-C; only lower metabolism in the cerebellum is being observed in MSA-C [162]. Presynaptic nigrostriatal dopamine transporter (DAT) imaging that has a high sensitivity for the diagnosis of PD shows variable results in MSA, PSP, and CBD (sensitivity 100%, 97.6%, 93.2%, and 60%, respectively), indicating that abnormal DAT scans may have a relatively low specificity in the differential diagnosis of APs [163]. ^18^F-fluoroethoxybenzovesamicol (FEOBV)-PET in REM sleep behavior disorder (RBD) from prodromal MSA shows increased uptake in specific brainstem nuclei, orbitofrontal, anterior cingulate, and paracentral cortex, suggesting a compensatory cholinergic upregulation in the initial phases of MSA [164]. Subcortical cholinergic dysfunction is more severe in MSA and PSP than in PD [165].

PSP had reduced acetylcholinesterase (AChE) activity in the paracentral region and the thalamus [166] and frequently shows frontal hypometabolism, although it varies in severity and pattern across PSP variants; it is common in PSP-SL, PSP-CBS, and PSP-F and least common in PSP-PGF, while PSP-SL and PSP-CBS show more severe hypometabolism than PSP-RS, PSP-P, and PSP-PGF. Hypometabolism in nearly all frontal regions, correlating with worse scores on the Frontal Assessment Battery, can separate PSP-SL and PSP-CBS variants [167], indicating that the cortical subtype with early prefrontal hypometabolism displays greater CI and DAT reduction than the brainstem subtype. Thus, disruption in dopaminergic cortico-basal ganglia pathways is crucial for the development of CI in PSP [168].

Recent studies found that the connectivity pattern of subcortical tau epicenters aligned with cortical perfusion patterns: cortical regions that are more closely connected to the tau epicenter show lower perfusion. This indicates that subcortical tau load is associated with remote perfusion reductions indicative of neuronal dysfunction in functionally connected cortical regions [169].

PSP shows significant locus ceruleus (LC) degeneration, highlighting the role of noradrenergic dysfunction associated with cognitive and behavioral features [170,171], while a reduction in muscarinic receptors in the mediodorsal thalamus and STN indicates their cholinergic dysfunction [148,172], although no significant changes in cortical cholinergic receptors were found in PSP [165]. GABA-A receptors decreased in metabolically affected cortical and subcortical regions in PSP [165].

In CBD, FDG-PET reveals asymmetric hypometabolism in the frontoparietal and occipital cortices with involvement of the ipsilateral subcortical structures, including basal ganglia and the thalamus [173]. This disease-specific metabolic brain network distinguishes CBD from other APs [174]. In CBD patients with CI, dysarthria and apraxia displayed significant hypometabolism in the parietal cortex, thalamus, and striatum contralateral to the more affected limbs [175]. It also involves frontoparietal regions, including the perirolandic area and ipsilateral basal ganglia and thalamus [176]. Verbal short-term memory is more impaired in left-handed CBS, and spatial attention more in the right-handed ones. Both of these functions reflect the functional specialization of both parietal pathways [177]. Patients with predominant dysarthria show left-sided hypometabolism in frontal regions (inferior frontal gyrus and premotor cortex) [87] and those with nfvPPA/CBD in the left fronto-insular and superior medial frontal cortex [178]. Semantic verbal fluency shows a positive correlation with FDG uptake in PET in the left frontal opercular and middle temporal gyri [89]. Ideomotor apraxia is associated with hypometabolism in the angular gyrus, and imitation apraxia with involvement of the postcentral gyrus, precuneus, and posterior cingulate gyrus. High variability in glucose metabolism and increased atrophy of the medial temporal lobe among patients with PSP and CBD correlate with worse cognitive performance independent of age and sex [179]. CBS showed decreased AChE activity in the postcentral region, frontal, parietal, and occipital cortex [166]. Both PSP and CBD show high glycemic variability in the medial temporal lobe [179]. In 4R-tauopathies, subcortical tau accumulation is linked to hypoperfusion in functionally connected cortical regions, suggesting that subcortical tau load may induce cortical dysfunction, which may contribute to clinical manifestations and heterogeneity [169].

#### 3.5.4. Morphological Differences and Overlaps Between APs

Despite the different backgrounds of the specific proteinopathies (MSA-synucleinopathy, PSP, and CBD 4R-tauopathies), the three APs show both morphological overlaps and differences. All of them present atrophy of wide GM and WM areas, with particular involvement of (pre)frontal and parietal cortices, striatum, thalamus, SN, and LC. MSA-P displays widespread volume loss in frontoparietal and striato-nigral areas, MSA-C in ponto-cerebellar systems and the middle cerebellar peduncle. PSP shows significant atrophy of the frontal region, striatum, thalamus, and midbrain, including SN, CBD in similar areas, and STN. In PSP, bilateral thalami and right putamen are smaller than in controls, not in MSA and PD, indicating morphologic and volumetric changes associated with PD and APs [180]. PSP shows degeneration of the middle cerebellar peduncle, which differs from MSA-P and PD [181]. PSP and PD share a similar GM reduction in the frontal lobe, while atrophy in the bilateral thalamus, insula, and brainstem is more severe in PSP, serving as a neuroanatomical marker for its differentiation [182]. MSA-CI shows more widespread impairment of hippocampal subfields compared with PD-CI, involving the trisynaptic loop and amygdala-hippocampus interaction. This may help to understand the differences in CI in MSA and PD [183]. In PSP and CBS, between-network connectivity components were identified that differed from HCs and were associated with variability in prognosis of both APs [156].

MSA-P exhibits symmetry in 15–35%, MSA-C in 40%, PSP-RS in about 84%, PSP-P in 53–55%, and PSP-CBS in less than 50%, whereas 90–99% of CBS cases show asymmetrical presentation [184]. FDG- and tau-PET demonstrate asymmetric hemispheric and subcortical changes contralateral to the clinically affected side [158].

Thalamus volume is smaller in PSP and CBD groups compared with PD. Disproportional involvement of the lateral thalamus in PSP and CBS, not observed in MSA and PD, could be a specific marker of tau-related neurodegeneration [185]. In PSP, thalamic involvement is diffuse and prevalent in its anterior part, whereas in CBD it is asymmetric and confined to the motor thalamus [186]. SN compacta is atrophic in MSA, PD, and PSP versus HCs; patients with PSP have greater SN reticulata reduction than those with MSA and PD, indicating different SN spatial lesion patterns between PSP and synucleinopathies, since MSA and PD show no SN reticulata changes [187]. Neuromelanin-sensitive MRI shows signal reduction in all parkinsonian syndromes, more severe in MSA and PSP than in PD without REM sleep behavior disorder; lower LC signal changes in PSP could partly be caused by the effect of age [188].

Other differences concern free water (FW) imaging: MSA shows changes in the cerebellum; PSP increased FW in supratentorial WM and midbrain. Together with PD, they show increased FW in SN, MSA, and PSP in the bilateral putamen; PD in the left GP externa and bilateral thalamus. Compared to HCs, MSA shows increased FW in the bilateral GP interna and left thalamus, while PSP displays increased FW of the right GP interna and externa and bilateral thalamus. These differences in the patterns of FW alterations between PD and APs may be used as markers for the diagnosis of these disorders [189]. Several studies showed greater putaminal iron deposition in PSP and MSA than in PD, which appears to be associated with increased disease severity and progression [190].

In MSA, strong FC between the orbitofrontal and temporal cortex, as well as changes in the limbic striato-thalamocortical and the prefrontal-cerebellar dentate nucleus circuits, are essential for the majority of neuropsychiatric disorders. In both PSP and MSA, widespread dysfunction of the corticostriatal circuit is evident. The majority of cortical dysfunctions are related to the involvement of cortico-subcortical circuits, in particular the cortico-basal ganglia-thalamocortical loops and the midbrain network. The CBS group shows more prominent asymmetry than the other AP groups, particularly in the perirolandic area, superior frontal gyrus, and anterior parietal lobe in early phases of disease. Striatal DAT uptake shows a significantly lower caudate-to-putamen uptake ratio in CBD, which differentiates it from PD in early disease [191]. In CBD, increased connectivity between DMN and attention networks is involved among multiple other neuronal networks [159]. PSP and CBD exhibit reduced FC between the lateral visual and auditory resting state networks, with PSP patients additionally showing lower FC between the cerebellar and insular resting state networks; these changes represent a higher degree of synchronization in damaged brain areas, while between-network resting state FC abnormalities may reflect degeneration of long-range WM fibers [192].

In all three APs, hypometabolism involves (pre)frontal and parietal regions, striatum, thalamus, and SN, although they differ in severity and pattern across PSP and CBD variants. In MSA, FDG-PET demonstrates characteristic nigrostriatal and olivocerebellar hypometabolism; in PSP, frontal lobe hypometabolism, and tau-PET confirms the underlying tauopathy.

The MDS criteria for PSP proposed a novel category, “probable 4R-tauopathy”, to address the phenotypic overlap between PSP and CBD. The CBS-4RT+ group, showing dysarthria and perseveration more often than the CBS-4RT– group, also shows prominent frontal hypometabolism and atrophy in the anterior cingulate and bilateral striata, but amyloid negativity [193]. Subcortical cholinergic dysfunction is more severe in MSA and PSP than in PD, while in PSP, noradrenergic dysfunction associated with LC degeneration is important.

### 3.6. Biomarkers for CI in APs

Prognostic biomarkers for CI in synucleinopathies include brain regional atrophy (MRI findings), metabolic and cerebral blood flow changes (FDG-PET), cortical rhythm slowing, cerebrospinal fluid (CSF) amyloid (Aβ-40), Val66Met polymorphism, APOE ε4 and ε2 alleles [194], GFAP and neurofilament light chain (NfL) levels in CSF and plasma [195], the latter being significantly increased in all cortical neurodegenerative disorders including APs [8,196]. Risk factors for cognitive decline in MSA and PSP include CSF signs of systemic inflammation [6,197]. Inflammatory biomarkers (SAA, CRP, IL-6, IL-8, and MCP-1) are increased in MSA compared to HCs and PD and correlate with more severe disease regarding CI (and motor symptoms), indicating an association between inflammation and a more aggressive disease course [198]. Early nigrosome changes, detected by neuromelanin-sensitive, diffusion-sensitive, and resting-state functional magnetic imaging measures, versus broader SN changes, may also serve as useful monitoring biomarkers for non-motor symptoms, such as CI [199]. Cystatin C is associated with cognitive decline in early-stage MSA [200]. Corticotropin-releasing hormone is decreased in Lewy body disease and other APs and correlated with CI and inflammation in αSyn-positive disorders [201]. DOPA decarboxylase in plasma is also increased in APs and can predict progression over a 3-year period [202], while LRRK2 in CSF is significantly increased in PD and APs with CI [203]. AD fluid markers (CSF p-tau, Aβ-40, and serum p-tau217) may modulate the clinical presentation of PSP/CBS [123], while tau-PET and plasma NfL show clinical progression of CI in patients with Aβ-negative CBS [204].

CSF p-tau198 is able to discriminate CI in PSP and CBD from HCs [205], while plasma NfL distinguishes PD from PSP and CBD and defines a distinct CBS-AD subtype [94]. PSP patients exhibit decreased levels of CSF-tau compared to HCs, while CBS cohorts present increased CSF-p-tau [206]. Lower levels of neuronal pentraxins and neurogranin in MSA and PSP, indicating synaptic dysfunction, may serve as biomarkers for CI progression in these disorders [207]. Tau quantification by real-time polymerase chain reaction (PCR) in skin lysates of PSP and CBD differentiated both from MSA (sensitivity 90%, specificity 86%), and its increase correlated with CI in both tauopathies [208].

A panel of plasma biomarkers for the differential diagnosis of APs includes a combination of αSyn, Aβ-40, Aβ-42, Aβ-42/40, and NfL; in terms of cognitive function, there was a relationship between plasma Aβ42/40 and MMSE score in patients with APs [209]. Plasma NfL and GFAP distinguished PSP from HC and from MSA-P [210], while plasma NfL, Aβ-42, and Aβ-40 could distinguish APs from PD and its subtypes [211].

### 3.7. Pathogenic Mechanisms

Whereas possible key genes (MAPT, APOE, APP, SNCA) and risk loci of APs have been identified [1,212,213,214], and recent research has unveiled essential basic mechanisms of neurodegeneration in MSA [215] and tauopathies [216,217], our knowledge about the pathogenesis of CI in this range of disorders is mainly dependent on recent neuroimaging findings, while concerning CI in APs, respective neuropathological data are rather poor.

The high prevalence of cognitive and neurobehavioral symptoms in MSA, according to animal models [218], is related to αSyn involvement of the amygdala, reflecting early changes in the corticolimbic system [219], while executive dysfunction, one of the earliest signs of CI, is linked to frontostriatal lesions, hypometabolism in the prefrontal cortex, and frontostriatal and fronto-cingulate WM lesions, all sequelae of αSyn pathology [27]. These and other lesions are related to neuro-inflammatory mechanisms, essential in the pathogenesis of MSA [197], due to activation of the complement pathway by aggregation of hyper-phosphorylated (p)αSyn [220], inducing microglial activation [215,221]. The resulting neuronal loss in cerebral GM (cortex, putamen, SN) and microlesions in cerebral WM are responsible for disruptions of essential neuronal networks. Hippocampal αSyn pathology with more neuronal cortical inclusions in the entorhinal cortex, cornu ammonis regions CA1-4, and subiculum correlates with memory impairment in MSA [42,222]. Furthermore, a greater burden of neuronal cytoplasmic inclusions in the limbic region is associated with CI [222]. The hippocampal subtype with dense neuronal cytoplasmic inclusions and severe neuronal loss in hippocampal CA1, subiculum, amygdala, and parahippocampal gyrus is a rare variant of MSA associated with CI [223,224]. MSA-specific αSyn strains, being extremely potent in inducing spreading, neuroinflammation, and degeneration, reflect the aggressive nature of this disease [225], and the recent demonstration of GCI-like fibrils within dark microglia suggests that they contribute to the origin of the spread of p-αSyn, which may be important for the pathogenesis of this disease [226].

In PSP and CBD, the contribution of p-tau pathology to alterations in neurons and glia leading to synaptic loss and disturbance of connections between neuronal circuits essential for cognitive functions (mainly prefrontal-limbic and multi-regional networks) is well established [124,217,227]. These neurodegenerative changes are associated with neuroinflammation [204]. Recent studies emphasized neuroinflammation mediated by T-cells in SN compacta of PSP and substantial loss of dopaminergic neurons in both MSA and PSP [228]. Combined PET studies indexing microglial activation and tau pathology demonstrated that both co-localize in the same brain regions [229], enhancing a pathological cascade leading to dysfunction of essential neuronal circuitries. In patients with 4R-tauopathies, tau-PET in basal ganglia and midbrain regions is negatively associated with striatal DAT availability, whereas those with α-synucleinopathies like MSA showed no such relationship, indicating that tau burden in brain regions involved in dopaminergic pathways is associated with aggravated dopaminergic dysfunction in tauopathies that might involve various functions [230]. Pathological tau in astrocytes and microglia [217], leading to neuronal loss and disconnection of neuronal circuits, can be considered as a morphological substrate of CI in PSP [227]; it is also associated with dopaminergic neuron loss in SN of MSA and PSP [228]. The spatial expansion of microglial activities parallels tau distribution across brain regions that are functionally connected to each other, suggesting that tau and inflammation are closely involved in the pathogenesis of 4R-tauopathies [229]. Neuronal-to-astrocytic plaque ratios in frontal cortex and basal ganglia in end-stage CBD were 12 times greater than in preclinical forms, suggesting that CBD may start as an astrogliopathy-inducing neuronal loss [231].

In CBD, like in PSP, the pattern of p-tau pathology is essential for the dysfunction of prefronto-subcortical and monoaminergic brainstem circuitries that are responsible for the development of CI and other comorbidities.

CBS has higher [18F]AV-1451 uptake (demonstrating AD-tau pathology), GM volume loss. and reduced synaptic density, the latter being more severe and widespread in the Aβ-negative group, while asymmetric synapse loss is in line with the clinically most affected side. Distinct patterns of tau pathology and reduced synaptic density indicate differences in the pathogenic mechanisms of CBS according to whether it is associated with the presence of AD or not. This is important for different therapeutic strategies in CBS [136]. αSyn and tau are proteins prone to pathological misfolding and aggregation that are normally found in the presynaptic and axonal compartments of neurons. Both are intrinsically disordered proteins, lacking stable secondary and tertiary structures. Misfolding initiates a homo-oligomerization and aggregation cascade culminating in the deposition of aggregated αSyn and tau in insoluble intracytoplasmic inclusions in many neurodegenerative diseases. There is significant overlap and co-occurrence of αSyn and tau pathologies; both can interact in cells, and their pathological conformations are capable of templating further misfolding and aggregation of each other [232]. The co-occurrence of tau with both soluble and insoluble αSyn is more likely to occur through monomer–fiber binding interactions, rather than monomer–monomer or co-aggregation [233]. The direct interaction of αSyn with tau is considered to promote the fibrillation of each of the proteins in vitro and in vivo [234].

Both αSyn and tau pathologies, inducing cell toxicity, neuro-inflammation, oxidative stress, and other complex pathogenic mechanisms, cause disruption of the relevant brain networks [235,236,237]. Thus, CI in APs can be suggested to be the result of neuronal network disorders due to the interaction of multiple pathological proteins [121].

### 3.8. Management Options

While there is no treatment to stop or retard the development of neurodegeneration in APs, a range of pharmacological, psychotherapeutic, passive immune, or brain stimulation therapies; cognitive training; rehabilitation methods; and other non-pharmacological treatments have been studied and/or are used for managing CI in APs, but appropriate therapies of CI are critical for comprehensive care of these parkinsonian disorders [238]. CI in MSA may benefit from optimization of dopamine and dopamine agonist therapy; the behavioral and cognitive effects of transdermal rotigotine (non-ergot dopamine agonist) are questionable [239]. Lithium that has shown positive functions in AD did not show benefits in MSA [240]. The efficacy of transnasal insulin for CI in MSA needs further confirmation [241]. Some effects of non-invasive brain stimulation (repetitive transcranial magnetic stimulation (rTMS) and transcranial direct current stimulation(tDCS)) on improving cognitive function in MSA also deserve further investigation [242]. Alternatively, cognitive behavioral therapy and cognitive rehabilitation have been found to improve specific cognitive deficits in MSA (and PD), but not all cognitive domains may benefit from this method that should be tailored to the patient’s specific impairments.

While a multitude of ongoing disease-modifying pharmacotherapies targeting tau in PSP, including genetic, microtubule-stabilizing, anti-phosphorylation and acetylation agents, anti-aggregants, and protein removal. Antioxidant, neuronal, and synaptic growth promotion therapies are in the pipeline [243,244]; specific pharmacological treatments are complemented by non-pharmacological managements. Levodopa has been shown to modulate semantic fluency in early stages of PSP-RS [245], and intrajejunal levodopa infusions have provided persistent improvement in some non-motor symptoms [246], although side effects of levodopa can worsen cognition in PSP [247]. Amantadin can improve some cognitive aspects, including alertness, but may also cause hallucinations and insomnia; anticholinergic drugs should be selected carefully to limit cognitive side effects [247]. Both tDCS and rTMS have beneficial effects on cognitive dysfunction in PSP [248,249].

According to a consensus statement of the US CurePSP Centers of Care [250], the following managements of CI in PSP/CBS are recommended: Pharmacological treatment includes cholinesterase, such as donepezil that should be discontinued if no benefit is observed. Non-pharmacological methods include caregiver and family education (adapt and emphasize activities); occupational therapy, compensatory technologies, and lifestyle modifications. For speech impairment in PSP/CBD, a number of methods to improve speech apraxia, non-fluent aphasia, and other speech disorders are recommended. Potential cognitive benefits have been reported for combined non-invasive neuromodulation [251] and rTMS [74]. In conclusion, AP patients can benefit from a multidisciplinary therapeutic approach with a person-specific combination of multiple modalities that should be screened and validated in order to obtain the best possible results.

### 3.9. Conclusions and Outlook

AP syndromes (MSA, PSP, and CBD), in addition to a combination of parkinsonism and motor/non-motor and other symptoms, are clinically characterized by a heterogenous spectrum of cognitive deficits that, despite differences in their basic pathologies (α-synucleinopathy and 4R tauopathies), show both overlaps and diversities, PSP and CBD generally being more severely impaired than MSA. While cognitive deficits in all three disorders manifest early in the course of the disease, their development is usually independent of the course and progression of motor and other symptoms. In MSA, CI differs in various domains between MSA-P and MSA-C, whereas the PSP and CBD groups show considerable differences in cognitive deficits among the clinical and morphological phenotypes, some of them mimicking AD and/or FTLD. As with the other clinical signs and symptoms, cognitive dysfunctions in APs reflect the different patterns of underlying brain pathologies that are induced by the deposition of pathological proteins in various structural elements of the brain. CI in MSA is one extreme, with the least impaired cognitive profile affecting executive, attentional, and visuospatial functions to PSP with much greater decline in global cognitive and executive functioning, episodic memory, attention and language deficits similar to CBD, where involvement of the majority of cognitive domains, including attention, executive and visuospatial functions, and language performance, are associated with speech apraxia and agraphia. Despite the different basic mechanisms of neurodegeneration that are responsible for the specific cognitive dysfunctions, many of the resulting brain lesions are similar in extent and severity, with variable involvement of cortical and subcortical areas. They cause complex dysfunction of multiple neuronal networks. CI in MSA is related to disruption of cortico-striato-limbic networks, that in PSP to disorders of fronto-striatal and cortico-basal ganglia/midbrain-cortical connections, while CI in CBD is related to asymmetric involvement of multiple circuitries, including default mode and attentional ones.

In all three APs, putative pathogenic factors for clinical symptoms, including CI, are deposition and spreading of the relevant pathological proteins (αSyn and p-tau), neuroinflammation, oxidative stress, and other complex molecular mechanisms, not all of which have been elucidated, but some of them can be detected by biomarkers that may become of diagnostic value.

In order to promote a more accurate and earlier diagnosis of CI in APs, clinical appreciation of “red flags”, together with standard criteria, should be considered in each of the APs, and evaluation of the neuropsychological profiles of the different types of APs and new, in particular, more specific biomarkers will be necessary. In addition to CSF and neuroimaging markers, new specific blood/serum markers should be found that may enable a more accurate and earlier non-invasive diagnosis of APs and their cognitive disorders. Neuropsychological tests should be used more intensively to evaluate general cognition, verbal and visual memory, working memory, constructive abilities, visuospatial skills, language, and executive functions in order to enable specific, personalized, and earlier management options. A detailed evaluation of the neuropsychological profiles of the different APs and their phenotypes and—if possible—the correlation with future biomarkers is necessary in order to detect specific deficits that should be considered as the basis of patient-specific treatments in order to increase the quality of life of both patients and caregivers.

Many of the cognitive dysfunctions, although being important for the quality of life of patients and caregivers, are still underdiagnosed. While no disease-stopping or retarding treatments for APs are available, a range of pharmacological and non-pharmacological methods, including non-invasive neuromodulation (rTMS and tDCS), behavioral, speech, and other treatment modalities, should be applied in a personalized manner in order to obtain reasonable results. In view of the heterogeneity of cognitive dysfunctions in APs and the limited objective findings in these highly complex disorders, further multidisciplinary studies are warranted in order to obtain better insight into their pathogenesis as a basis for the development of further diagnostic biomarkers and future adequate treatment modalities of these debilitating comorbidities.

## Figures and Tables

**Table 3:** Neuroimaging findings associated with CI in MSA: functional MRI.

Findings	References
Dysfunction of fronto-striatal connections	[100]
Disruption of the dorsolateral prefrontal-insular network	[102]
Decreased FC, right dental nucleus—cerebellum	[140]
Decreased FC, putamen and cerebellum—cortex	[108]
Decreased FC, cerebello-prefrontal network	[17,141]
Decreased FC, cerebello-amygdala network	
Decreased FC, DLPFC—cerebellum	[142]
Decreased FC, DLPFC—inferior parietal lobe	
Decreased FC, DLPFC—dentate nucleus	[143]
Enhanced FC, dentate nucleus—posterior cingulate cortex	
Enhanced FC, cerebellum—frontoparietal network	[144]
Enhanced FC in the prefrontal default mode network	
Enhanced FC in the ventral attention network	

CI: cognitive impairment; MSA: multiple system atrophy; FC: functional connectivity; DLPFC: dorsolateral prefrontal cortex.

**Table 5:** Neuroimaging findings associated with CI in CBD: functional MRI.

Findings	References
Reduced FC, bilateral dorsal attentional network	[154]
Reduced FC between dorsal, attentional, and motor networks	[156]
Reduced FC within each of the dorsal, attentional, and motor networks	[156]
Reduced FC in right operculum, medial superior-frontal gyrus, and bilateral caudate nuclei	[126]
Increased FC in anterior cingulum, medial superior-frontal gyrus, and bilateral caudate nuclei	[126]
Reduced FC, dorsal attentional network	[155]
Reduced FC, thalamus-motor cortices	[157]
Reduced FC, thalamic network	[123]

CI: cognitive impairment; CBD: corticobasal degeneration; FC: functional connectivity.

## Abbreviations

PSP	progressive supranuclear palsy
nfvPPA	PSP with nonfluent/agrammatic primary progressive aphasia
PSP-C	PSP with cerebellar ataxia
PSP-CBS	PSP with predominant corticobasal syndrome
PSP-F	PSP with predominant frontal/cognitive presentation
PSP-OM	PSP with predominant oculomotor dysfunction
PSP-P	PSP with predominant parkinsonism
PSP-PGF	PSP with predominant gait freezing and motor blocks
PSP-PLS	PSP with primary lateral sclerosis
PSP-RS	Richardson’s syndrome/classic PSP
PSP-SL	PSP with predominant speech/language disorder
AD	Alzheimer disease
AP	atypical parkinsonism
αSyn	α-synuclein
CBD	corticobasal degeneration
CBS	corticobasal syndrome
CI	cognitive impairment
CSF	cerebrospinal fluid
DAN	dorsal attentional network
DAT	dopamine transporter
DLPFC	dorsolateral prefrontal cortex
DMN	default mode network
EF	executive function
FC	functional connectivity
FTLD	frontotemporal lobe degeneration
FW	free water
GCI	glial cytoplasmic inclusion
GM	gray matter
GP	globus pallidus
HCs	healthy controls
LC	locus ceruleus
MDS	Movement Disorder Society
MSA	multiple system atrophy
MSA-C	MSA cerebellar variant
MSA-P	MSA parkinsonism variant
NCI	neuronal cortical inclusion
PD	Parkinson’s disease
PPA	primary progressive aphasia
QoL	quality of life
rTMS	repetitive transcranial magnetic stimulation
SN	substantia nigra
STN	subthalamic nucleus
tDCS	transcranial direct current stimulation
WM	white matter

## References

[1] Pasqualotto A., da Silva V., Pellenz F.M., Schuh A.F.S., Schwartz I.V.D., Siebert M. (2024). Identification of metabolic pathways and key genes associated with atypical parkinsonism using a systems biology approach. Metab. Brain Dis..

[2] Wittke C., Petkovic S., Dobricic V., Schaake S., Respondek G., Weissbach A., Madoev H., Trinh J., Vollstedt E.J., Kuhnke N. (2021). Genotype-phenotype relations for the atypical parkinsonism genes: MDSGene systematic review. Mov. Disord..

[3] Stamelou M., Bhatia K.P. (2015). Atypical parkinsonism: Diagnosis and treatment. Neurol. Clin..

[4] Belvisi D., Berardelli I., Suppa A., Fabbrini A., Pasquini M., Pompili M., Fabbrini G. (2018). Neuropsychiatric disturbances in atypical parkinsonian disorders. Neuropsychiatr. Dis. Treat..

[5] Vertes A.C., Beato M.R., Sonne J., Khan Suheb M.Z. (2023). Parkinson-Plus Syndrome. StatPearls [Internet].

[6] Bäckström D., Granåsen G., Mo S.J., Riklund K., Trupp M., Zetterberg H., Blennow K., Forsgren L., Domellöf M.E. (2022). Prediction and early biomarkers of cognitive decline in Parkinson disease and atypical parkinsonism: A population-based study. Brain Commun..

[7] Raimo S., Gaita M., Cropano M., Mautone G., D’Iorio A., Trojano L., Santangelo G. (2023). The cognitive profile of atypical parkinsonism: A meta-analysis. Neuropsychol. Rev..

[8] Giannakis A., Sioka C., Kloufetou E., Konitsiotis S. (2024). Cognitive impairment in Parkinson’s disease and other parkinsonian syndromes. J. Neural Transm..

[9] Jellinger K.A. (2023). Pathomechanisms of depression in progressive supranuclear palsy. J. Neural Transm..

[10] Jellinger K.A. (2023). Pathomechanisms of depression in multiple system atrophy. J. Neural Transm..

[11] Jellinger K.A. (2024). The enigma of depression in corticobasal degeneration, a frequent but poorly understood co-morbidity. J. Neural Transm..

[12] Jellinger K.A. (2023). Mild cognitive impairment in multiple system atrophy: A brain network disorder. J. Neural Transm..

[13] Jellinger K.A. (2022). Morphological basis of Parkinson disease-associated cognitive impairment: An update. J. Neural Transm..

[14] Jellinger K.A. (2024). Mild cognitive impairment in Parkinson’s disease: Current view. Front. Cogn..

[15] Gilman S., Wenning G.K., Low P.A., Brooks D.J., Mathias C.J., Trojanowski J.Q., Wood N.W., Colosimo C., Durr A., Fowler C.J. (2008). Second consensus statement on the diagnosis of multiple system atrophy. Neurology.

[16] Stankovic I., Krismer F., Jesic A., Antonini A., Benke T., Brown R.G., Burn D.J., Holton J.L., Kaufmann H., Kostic V.S. (2014). Cognitive impairment in multiple system atrophy: A position statement by the neuropsychology task force of the MDS multiple system atrophy (MODIMSA) study group. Mov. Disord..

[17] Zhang L., Xue F., Yue K., Peng H., Wu Y., Sha O., Yang L., Ding Y. (2018). Brain morphological alteration and cognitive dysfunction in multiple system atrophy. Quant. Imaging Med. Surg..

[18] Wenning G.K., Tison F., Ben Shlomo Y., Daniel S.E., Quinn N.P. (1997). Multiple system atrophy: A review of 203 pathologically proven cases. Mov. Disord..

[19] Jellinger K.A. (2023). Morphological differences between the two major subtypes of multiple system atrophy with cognitive impairment. Park. Relat. Disord..

[20] Koga S., Cheshire W.P., Tipton P.W., Driver-Dunckley E.D., Wszolek Z.K., Uitti R.J., Graff-Radford N.R., van Gerpen J.A., Dickson D.W. (2021). Clinical features of autopsy-confirmed multiple system atrophy in the Mayo Clinic Florida brain bank. Park. Relat. Disord..

[21] Cui Y., Cao S., Li F., Feng T. (2022). Prevalence and clinical characteristics of dementia and cognitive impairment in multiple system atrophy: A systematic review and meta-analysis. J. Park. Dis..

[22] Abrahão A., Dutra L.A., Braga Neto P., Pedroso J.L., de Oliveira R.A., Barsottini O.G.P. (2011). Cognitive impairment in multiple system atrophy: Changing concepts. Dement. Neuropsychol..

[23] Brown R.G., Lacomblez L., Landwehrmeyer B.G., Bak T., Uttner I., Dubois B., Agid Y., Ludolph A., Bensimon G., Payan C. (2010). Cognitive impairment in patients with multiple system atrophy and progressive supranuclear palsy. Brain.

[24] Hatakeyama M., Sato T., Takahashi T., Kanazawa M., Onodera O., Nishizawa M., Shimohata T. (2018). Predictors of cognitive impairment in multiple system atrophy. J. Neurol. Sci..

[25] Eschlböck S., Delazer M., Krismer F., Bodner T., Fanciulli A., Heim B., Heras Garvin A., Kaindlstorfer C., Karner E., Mair K. (2020). Cognition in multiple system atrophy: A single-center cohort study. Ann. Clin. Transl. Neurol..

[26] Nasri A., Gharbi A., Sghaier I., Mrabet S., Souissi A., Gargouri A., Djebara M.B., Kacem I., Gouider R. (2022). Determinants of cognitive impairment in multiple system atrophy: Clinical and genetic study. PLoS ONE.

[27] Kübler D., Kobylecki C., McDonald K.R., Anton-Rodriguez J.M., Herholz K., Carter S.F., Hinz R., Thompson J.C., Al-Fatly B., Gerhard A. (2023). Structural and metabolic correlates of neuropsychological profiles in multiple system atrophy and Parkinson’s disease. Park. Relat. Disord..

[28] Santangelo G., Cuoco S., Picillo M., Erro R., Squillante M., Volpe G., Cozzolino A., Cicarelli G., Barone P., Pellecchia M.T. (2020). Evolution of neuropsychological profile in motor subtypes of multiple system atrophy. Park. Relat. Disord..

[29] Santangelo G., Cuoco S., Picillo M., Erro R., Squillante M., Volpe G., Cozzolino A., Cicarelli G., Barone P., Pellecchia M.T. (2020). Theory of Mind in multiple system atrophy: Comparison with Parkinson’s disease and healthy subjects. J. Neural Transm..

[30] Siri C., Duerr S., Canesi M., Delazer M., Esselink R., Bloem B.R., Gurevich T., Balas M., Giladi N., Santacruz P. (2013). A cross-sectional multicenter study of cognitive and behavioural features in multiple system atrophy patients of the parkinsonian and cerebellar type. J. Neural Transm..

[31] Chang C.C., Chang Y.Y., Chang W.N., Lee Y.C., Wang Y.L., Lui C.C., Huang C.W., Liu W.L. (2009). Cognitive deficits in multiple system atrophy correlate with frontal atrophy and disease duration. Eur. J. Neurol..

[32] Lazzeri G., Franco G., Difonzo T., Carandina A., Gramegna C., Vergari M., Arienti F., Naci A., Scatà C., Monfrini E. (2022). Cognitive and autonomic dysfunction in multiple system atrophy type P and C: A comparative study. Front. Neurol..

[33] Balas M., Balash Y., Giladi N., Gurevich T. (2010). Cognition in multiple system atrophy: Neuropsychological profile and interaction with mood. J. Neural Transm..

[34] Gatto E., Demey I., Sanguinetti A., Parisi V., Etcheverry J.L., Rojas G., Wenning G.K. (2014). Cognition in a multiple system atrophy series of cases from Argentina. Arq. Neuropsiquiatr..

[35] Kawai Y., Suenaga M., Takeda A., Ito M., Watanabe H., Tanaka F., Kato K., Fukatsu H., Naganawa S., Kato T. (2008). Cognitive impairments in multiple system atrophy: MSA-C vs MSA-P. Neurology.

[36] Barcelos L.B., Saad F., Giacominelli C., Saba R.A., de Carvalho Aguiar P.M., Silva S.M.A., Borges V., Bertolucci P.H.F., Ferraz H.B. (2018). Neuropsychological and clinical heterogeneity of cognitive impairment in patients with multiple system atrophy. Clin. Neurol. Neurosurg..

[37] Dash S., Mahale R., Netravathi M., Kamble N.L., Holla V., Yadav R., Pal P.K. (2022). Cognition in patients with multiple system atrophy (MSA) and its neuroimaging correlation: A prospective case-control study. Cureus.

[38] Ruiz Barrio I., Miki Y., Jaunmuktane Z.T., Warner T., De Pablo-Fernandez E. (2023). Association between orthostatic hypotension and dementia in patients with parkinson disease and multiple system atrophy. Neurology.

[39] Yang X.L., Guo Y., Chen S.F., Cui M., Shao R.R., Huang Y.Y., Luo Y.F., Dong Z.Y., Dong Q., Wu D.H. (2023). Cerebral small vessel disease is associated with motor, cognitive, and emotional dysfunction in multiple system atrophy. J. Park. Dis..

[40] Zhang L., Hou Y., Cao B., Wei Q.Q., Ou R., Liu K., Lin J., Yang T., Xiao Y., Zhao B. (2021). Vascular risk factors and cognition in multiple system atrophy. Front. Neurosci..

[41] Sekiya H., Koga S., Murakami A., DeTure M., Ross O.A., Uitti R.J., Cheshire W.P., Wszolek Z.K., Dickson D.W. (2024). Frequency of comorbid pathologies and their clinical impact in multiple system atrophy. Mov. Disord..

[42] Miki Y., Foti S.C., Hansen D., Strand K.M., Asi Y.T., Tsushima E., Jaunmuktane Z., Lees A.J., Warner T.T., Quinn N. (2020). Hippocampal a-synuclein pathology correlates with memory impairment in multiple system atrophy. Brain.

[43] Homma T., Mochizuki Y., Komori T., Isozaki E. (2016). Frequent globular neuronal cytoplasmic inclusions in the medial temporal region as a possible characteristic feature in multiple system atrophy with dementia. Neuropathology.

[44] Saito M., Hara M., Ebashi M., Morita A., Okada K., Homma T., Sugitani M., Endo K., Uchihara T., Kamei S. (2017). Perirhinal accumulation of neuronal alpha-synuclein in a multiple system atrophy patient with dementia. Neuropathology.

[45] Asi Y.T., Ling H., Ahmed Z., Lees A.J., Revesz T., Holton J.L. (2014). Neuropathological features of multiple system atrophy with cognitive impairment. Mov. Disord..

[46] Tanaka M.T., Tanji K., Miki Y., Ozaki T., Mori F., Hayashi H., Kakita A., Wakabayashi K. (2022). Phosphorylation of tau at threonine 231 in patients with multiple system atrophy and in a mouse model. J. Neuropathol. Exp. Neurol..

[47] Terni B., Rey M.J., Boluda S., Torrejon-Escribano B., Sabate M.P., Calopa M., van Leeuwen F.W., Ferrer I. (2007). Mutant ubiquitin and p62 immunoreactivity in cases of combined multiple system atrophy and Alzheimer’s disease. Acta Neuropathol..

[48] Gerstenecker A., Mast B., Duff K., Ferman T.J., Litvan I. (2013). Executive dysfunction is the primary cognitive impairment in progressive supranuclear palsy. Arch. Clin. Neuropsychol..

[49] Höglinger G.U., Respondek G., Stamelou M., Kurz C., Josephs K.A., Lang A.E., Mollenhauer B., Müller U., Nilsson C., Whitwell J.L. (2017). Clinical diagnosis of progressive supranuclear palsy: The movement disorder society criteria. Mov. Disord..

[50] Boxer A.L., Yu J.T., Golbe L.I., Litvan I., Lang A.E., Höglinger G.U. (2017). Advances in progressive supranuclear palsy: New diagnostic criteria, biomarkers, and therapeutic approaches. Lancet Neurol..

[51] Nasri A., Sghaier I., Neji A., Gharbi A., Abida Y., Mrabet S., Gargouri A., Djebara M.B., Kacem I., Gouider R. (2024). Phenotypic spectrum of progressive supranuclear palsy: Clinical study and apolipoprotein E effect. J. Mov. Disord..

[52] Coughlin D.G., Dickson D.W., Josephs K.A., Litvan I. (2021). Progressive supranuclear palsy and corticobasal degeneration. Adv. Exp. Med. Biol..

[53] Jellinger K.A. (2023). Pathomechanisms of cognitive impairment in progressive supranuclear palsy. J. Neural Transm..

[54] Bruns M.B., Josephs K.A. (2013). Neuropsychiatry of corticobasal degeneration and progressive supranuclear palsy. Int. Rev. Psychiatry.

[55] Painous C., Martí M.J., Simonet C., Garrido A., Valldeoriola F., Muñoz E., Cámara A., Compta Y. (2020). Prediagnostic motor and non-motor symptoms in progressive supranuclear palsy: The step-back PSP study. Park. Relat. Disord..

[56] Ichijo K., Takahata K., Kurose S., Watanabe T., Nagase Y., Endo H., Tagai K., Ishitobi M., Higuchi M. (2024). Late-life mood disorder as the initial presentation of progressive supranuclear palsy: A case series. PCN Rep..

[57] Campagnolo M., Weis L., Fogliano C., Cianci V., Garon M., Fiorenzato E., Carecchio M., Ferreri F., Bisiacchi P., Antonini A. (2023). Clinical, cognitive, and morphometric profiles of progressive supranuclear palsy phenotypes. J. Neural Transm..

[58] Millar D., Griffiths P., Zermansky A.J., Burn D.J. (2006). Characterizing behavioral and cognitive dysexecutive changes in progressive supranuclear palsy. Mov. Disord..

[59] Parthimos T.P., Schulpis K.H. (2020). The progressive supranuclear palsy: Past and present aspects. Clin. Gerontol..

[60] Luca A., Nicoletti A., Donzuso G., Terravecchia C., Cicero C.E., D’Agate C., Rascuná C., Manna R., Mostile G., Zappia M. (2021). Phonemic verbal fluency and midbrain atrophy in progressive supranuclear palsy. J. Alzheimers Dis..

[61] Josephs K.A., Duffy J.R. (2008). Apraxia of speech and nonfluent aphasia: A new clinical marker for corticobasal degeneration and progressive supranuclear palsy. Curr. Opin. Neurol..

[62] Lee Y.C., Williams D.R., Anderson J.F.I. (2018). Prospective characterization of cognitive function in typical and ’brainstem predominant’ progressive supranuclear palsy phenotypes. J. Mov. Disord..

[63] Kobylecki C., Jones M., Thompson J.C., Richardson A.M., Neary D., Mann D.M., Snowden J.S., Gerhard A. (2015). Cognitive-behavioural features of progressive supranuclear palsy syndrome overlap with frontotemporal dementia. J. Neurol..

[64] Duff K., McDermott D., Luong D., Randolph C., Boxer A.L. (2019). Cognitive deficits in progressive supranuclear palsy on the Repeatable Battery for the Assessment of Neuropsychological Status. J. Clin. Exp. Neuropsychol..

[65] Koga S., Parks A., Kasanuki K., Sanchez-Contreras M., Baker M.C., Josephs K.A., Ahlskog J.E., Uitti R.J., Graff-Radford N., van Gerpen J.A. (2017). Cognitive impairment in progressive supranuclear palsy is associated with tau burden. Mov. Disord..

[66] Giagkou N., Höglinger G.U., Stamelou M. (2019). Progressive supranuclear palsy. Int. Rev. Neurobiol..

[67] de Souza L.C., Bertoux M., Radakovic R., Hornberger M., Mariano L.I., Resende E.P.F., Quesque F., Guimarães H.C., Gambogi L.B., Tumas V. (2022). I’m looking through you: Mentalizing in frontotemporal dementia and progressive supranuclear palsy. Cortex.

[68] Macedo A.C., Mariano L.I., Martins M.I., Friedlaender C.V., Ventura J.M., de Faria Rocha J.V., Camargos S.T., Cardoso F.E.C., Caramelli P., de Souza L.C. (2022). Do patients with progressive supranuclear palsy have episodic memory impairment? A systematic review. Mov. Disord. Clin. Pract..

[69] Fiorenzato E., Antonini A., Camparini V., Weis L., Semenza C., Biundo R. (2019). Characteristics and progression of cognitive deficits in progressive supranuclear palsy vs. multiple system atrophy and Parkinson’s disease. J. Neural Transm..

[70] Mathew R., Bak T.H., Hodges J.R. (2011). Screening for cognitive dysfunction in corticobasal syndrome: Utility of Addenbrooke’s cognitive examination. Dement. Geriatr. Cogn. Disord..

[71] Armstrong M.J., Litvan I., Lang A.E., Bak T.H., Bhatia K.P., Borroni B., Boxer A.L., Dickson D.W., Grossman M., Hallett M. (2013). Criteria for the diagnosis of corticobasal degeneration. Neurology.

[72] Shir D., Thu Pham N.T., Botha H., Koga S., Kouri N., Ali F., Knopman D.S., Petersen R.C., Boeve B.F., Kremers W.K. (2023). Clinicoradiologic and neuropathologic evaluation of corticobasal syndrome. Neurology.

[73] Saranza G.M., Whitwell J.L., Kovacs G.G., Lang A.E. (2019). Corticobasal degeneration. Int. Rev. Neurobiol..

[74] Shehata H.S., Shalaby N.M., Esmail E.H., Fahmy E. (2015). Corticobasal degeneration: Clinical characteristics and multidisciplinary therapeutic approach in 26 patients. Neurol. Sci..

[75] Lee S.E., Rabinovici G.D., Mayo M.C., Wilson S.M., Seeley W.W., DeArmond S.J., Huang E.J., Trojanowski J.Q., Growdon M.E., Jang J.Y. (2011). Clinicopathological correlations in corticobasal degeneration. Ann. Neurol..

[76] Oliveira L.M., Barcellos I., Teive H.A.G., Munhoz R.P. (2017). Cognitive dysfunction in corticobasal degeneration. Arq. Neuropsiquiatr..

[77] Aiba I., Hayashi Y., Shimohata T., Yoshida M., Saito Y., Wakabayashi K., Komori T., Hasegawa M., Ikeuchi T., Tokumaru A.M. (2023). Clinical course of pathologically confirmed corticobasal degeneration and corticobasal syndrome. Brain Commun..

[78] Tse N.Y., Chen Y., Irish M., Cordato N.J., Landin-Romero R., Hodges J.R., Piguet O., Ahmed R.M. (2020). Cerebellar contributions to cognition in corticobasal syndrome and progressive supranuclear palsy. Brain Commun..

[79] Sakae N., Santos O.A., Pedraza O., Litvan I., Murray M.E., Duara R., Uitti R.J., Wszolek Z.K., Graff-Radford N.R., Josephs K.A. (2020). Clinical and pathologic features of cognitive-predominant corticobasal degeneration. Neurology.

[80] Kurihara M., Arakawa A., Tokumaru A.M., Matsubara T., Eguchi H., Shimo Y., Hasegawa M., Kanemaru K., Takeda K., Iwata A. (2024). Dynamic aphasia as an early sign of corticobasal degeneration: Clinico-radio-pathological correlation. eNeurologicalSci.

[81] Graham N.L., Bak T.H., Hodges J.R. (2003). Corticobasal degeneration as a cognitive disorder. Mov. Disord..

[82] de Brito-Marques P.R., Vieira-Mello R.J., Montenegro L., Aragão M.F.V. (2011). Clinicopathologic analysis of progressive non-fluent aphasia and corticobasal degeneration: Case report and review. Dement. Neuropsychol..

[83] Kertesz A., McMonagle P. (2010). Behavior and cognition in corticobasal degeneration and progressive supranuclear palsy. J. Neurol. Sci..

[84] Josephs K.A., Petersen R.C., Knopman D.S., Boeve B.F., Whitwell J.L., Duffy J.R., Parisi J.E., Dickson D.W. (2006). Clinicopathologic analysis of frontotemporal and corticobasal degenerations and PSP. Neurology.

[85] Josephs K.A., Duffy J.R., Strand E.A., Machulda M.M., Senjem M.L., Gunter J.L., Schwarz C.G., Reid R.I., Spychalla A.J., Lowe V.J. (2014). The evolution of primary progressive apraxia of speech. Brain.

[86] Duffy J.R. (2006). Apraxia of speech in degenerative neurologic disease. Aphasiology.

[87] Parmera J.B., de Almeida I.J., de Oliveira M.C.B., Silagi M.L., de Godoi Carneiro C., Studart-Neto A., Ono C.R., Reis Barbosa E., Nitrini R., Buchpiguel C.A. (2021). Metabolic and structural signatures of speech and language impairment in corticobasal syndrome: A multimodal PET/MRI study. Front. Neurol..

[88] Garcia-Guaqueta D.P., Stephens Y.C., Ali F., Utianski R.L., Duffy J.R., Clark H.M., Thu Pham N.T., Machulda M.M., Lowe V.J., Dickson D.W. (2024). Comparing classic-onset corticobasal syndrome to speech/language-onset corticobasal syndrome. Parkinsonism Relat. Disord..

[89] Parmera J.B., Coutinho A.M., Aranha M.R., Studart-Neto A., de Godoi Carneiro C., de Almeida I.J., Fontoura Solla D.J., Ono C.R., Barbosa E.R., Nitrini R. (2021). FDG-PET patterns predict amyloid deposition and clinical profile in corticobasal syndrome. Mov. Disord..

[90] Ruggeri M., Biagioli C., Ricci M., Gerace C., Blundo C. (2020). Progressive aphasia, apraxia of speech and agraphia in corticobasal degeneration: A 12-case series clinical and neuropsychological descriptive study. Int. J. Lang. Commun. Disord..

[91] Jia P., Zhang J., Han J., Ji Y. (2022). Clinical outcomes and cognitive impairments between progressive supranuclear palsy and multiple system atrophy. Brain Behav..

[92] Santos-Santos M.A., Mandelli M.L., Binney R.J., Ogar J., Wilson S.M., Henry M.L., Hubbard H.I., Meese M., Attygalle S., Rosenberg L. (2016). Features of patients with nonfluent/agrammatic primary progressive aphasia with underlying progressive supranuclear palsy pathology or corticobasal degeneration. JAMA Neurol..

[93] Jellinger K.A. (2023). Pathomechanisms of cognitive and behavioral impairment in corticobasal degeneration. J. Neural Transm..

[94] Jabbari E., Holland N., Chelban V., Jones P.S., Lamb R., Rawlinson C., Guo T., Costantini A.A., Tan M.M.X., Heslegrave A.J. (2020). Diagnosis across the spectrum of progressive supranuclear palsy and corticobasal syndrome. JAMA Neurol..

[95] Respondek G., Kurz C., Arzberger T., Compta Y., Englund E., Ferguson L.W., Gelpi E., Giese A., Irwin D.J., Meissner W.G. (2017). Which ante mortem clinical features predict progressive supranuclear palsy pathology?. Mov. Disord..

[96] Jensen I., Heine J., Ruf V.C., Compta Y., Porcel L.M., Troakes C., Vamanu A., Downes S., Irwin D., Cohen J. (2024). Impact of magnetic resonance imaging markers on the diagnostic performance of the International Parkinson and Movement Disorder Society Multiple System Atrophy criteria. Mov. Disord..

[97] Krismer F., Péran P., Beliveau V., Seppi K., Arribarat G., Pavy-Le Traon A., Meissner W.G., Foubert-Samier A., Fabbri M., Schocke M.M. (2024). Progressive brain atrophy in multiple system atrophy: A longitudinal, multicenter, magnetic resonance imaging study. Mov. Disord..

[98] Cao B., Zhao B., Wei Q.Q., Chen K., Yang J., Ou R., Wu Y., Shang H.F. (2015). The global cognition, frontal lobe dysfunction and behavior changes in chinese patients with multiple system atrophy. PLoS ONE.

[99] Caso F., Canu E., Lukic M.J., Petrovic I.N., Fontana A., Nikolic I., Kostic V.S., Filippi M., Agosta F. (2020). Cognitive impairment and structural brain damage in multiple system atrophy-parkinsonian variant. J. Neurol..

[100] Fiorenzato E., Weis L., Seppi K., Onofrj M., Cortelli P., Zanigni S., Tonon C., Kaufmann H., Shepherd T.M., Poewe W. (2017). Brain structural profile of multiple system atrophy patients with cognitive impairment. J. Neural Transm..

[101] Breukelaar I.A., Antees C., Grieve S.M., Foster S.L., Gomes L., Williams L.M., Korgaonkar M.S. (2017). Cognitive control network anatomy correlates with neurocognitive behavior: A longitudinal study. Hum. Brain Mapp..

[102] Yang H., Luo X., Yu H., Guo M., Cao C., Li Y., Fan G. (2020). Altered resting-state voxel-level whole-brain functional connectivity in multiple system atrophy patients with cognitive impairment. Clin. Neurophysiol..

[103] Hara K., Watanabe H., Bagarinao E., Kawabata K., Yoneyama N., Ohdake R., Imai K., Masuda M., Yokoi T., Ogura A. (2018). Corpus callosal involvement is correlated with cognitive impairment in multiple system atrophy. J. Neurol..

[104] Kiernan J.A. (2012). Anatomy of the temporal lobe. Epilepsy Res. Treat..

[105] Fan J., Zhong M., Gan J., Liu W., Niu C., Liao H., Zhang H., Tan C., Yi J., Zhu X. (2017). Spontaneous neural activity in the right superior temporal gyrus and left middle temporal gyrus is associated with insight level in obsessive-compulsive disorder. J. Affect. Disord..

[106] Wei T., Liang X., He Y., Zang Y., Han Z., Caramazza A., Bi Y. (2012). Predicting conceptual processing capacity from spontaneous neuronal activity of the left middle temporal gyrus. J. Neurosci..

[107] Sugiyama A., Yokota H., Hirano S., Wang J., Ito S., Kuwabara S. (2023). Association between cognitive impairment and hippocampal subfield volumes in multiple system atrophy. Park. Dis..

[108] Cuoco S., Ponticorvo S., Bisogno R., Manara R., Esposito F., Di Salle G., Di Salle F., Amboni M., Erro R., Picillo M. (2022). Magnetic resonance T1w/T2w ratio in the putamen and cerebellum as a marker of cognitive impairment in MSA: A longitudinal study. Cerebellum.

[109] Nicastro N., Rodriguez P.V., Malpetti M., Bevan-Jones W.R., Simon Jones P., Passamonti L., Aigbirhio F.I., O’Brien J.T., Rowe J.B. (2020). (18)F-AV1451 PET imaging and multimodal MRI changes in progressive supranuclear palsy. J. Neurol..

[110] Stezin A., Lenka A., Jhunjhunwala K., Saini J., Pal P.K. (2017). Advanced structural neuroimaging in progressive supranuclear palsy: Where do we stand?. Park. Relat. Disord..

[111] Pan P., Liu Y., Zhang Y., Zhao H., Ye X., Xu Y. (2017). Brain gray matter abnormalities in progressive supranuclear palsy revisited. Oncotarget.

[112] Scotton W.J., Bocchetta M., Todd E., Cash D.M., Oxtoby N., VandeVrede L., Heuer H., Alexander D.C., Rowe J.B., Morris H.R. (2022). A data-driven model of brain volume changes in progressive supranuclear palsy. Brain Commun..

[113] Howard E., Ballinger S., Kinney N.G., Balgenorth Y., Ehrhardt A., Phillips J.S., Irwin D.J., Grossman M., Cousins K.A.Q. (2022). Frontal atrophy and executive dysfunction relate to complex numbers impairment in progressive supranuclear palsy. J. Alzheimers Dis..

[114] Agosta F., Galantucci S., Svetel M., Lukic M.J., Copetti M., Davidovic K., Tomic A., Spinelli E.G., Kostic V.S., Filippi M. (2014). Clinical, cognitive, and behavioural correlates of white matter damage in progressive supranuclear palsy. J. Neurol..

[115] Cordato N.J., Duggins A.J., Halliday G.M., Morris J.G., Pantelis C. (2005). Clinical deficits correlate with regional cerebral atrophy in progressive supranuclear palsy. Brain.

[116] Sintini I., Schwarz C.G., Senjem M.L., Reid R.I., Botha H., Ali F., Ahlskog J.E., Jack C.R., Lowe V.J., Josephs K.A. (2019). Multimodal neuroimaging relationships in progressive supranuclear palsy. Park. Relat. Disord..

[117] Uchida W., Kamagata K., Andica C., Takabayashi K., Saito Y., Owaki M., Fujita S., Hagiwara A., Wada A., Akashi T. (2023). Fiber-specific micro- and macroscopic white matter alterations in progressive supranuclear palsy and corticobasal syndrome. NPJ Park. Dis..

[118] Chung E.J., Cho H.J., Jang W., Hur D.Y., Kim Y.S., Lee K.H., Kim S.J. (2022). A case of pathologically confirmed corticobasal degeneration initially presenting as progressive supranuclear palsy syndrome. J. Korean Med. Sci..

[119] Di Stasio F., Suppa A., Marsili L., Upadhyay N., Asci F., Bologna M., Colosimo C., Fabbrini G., Pantano P., Berardelli A. (2019). Corticobasal syndrome: Neuroimaging and neurophysiological advances. Eur. J. Neurol..

[120] Huey E.D., Goveia E.N., Paviol S., Pardini M., Krueger F., Zamboni G., Tierney M.C., Wassermann E.M., Grafman J. (2009). Executive dysfunction in frontotemporal dementia and corticobasal syndrome. Neurology.

[121] Constantinides V.C., Paraskevas G.P., Paraskevas P.G., Stefanis L., Kapaki E. (2019). Corticobasal degeneration and corticobasal syndrome: A review. Clin. Park. Relat. Disord..

[122] Josephs K.A., Duffy J.R., Strand E.A., Machulda M.M., Senjem M.L., Master A.V., Lowe V.J., Jack C.R., Whitwell J.L. (2012). Characterizing a neurodegenerative syndrome: Primary progressive apraxia of speech. Brain.

[123] Garcia-Cordero I., Anastassiadis C., Khoja A., Morales-Rivero A., Thapa S., Vasilevskaya A., Davenport C., Sumra V., Couto B., Multani N. (2024). Evaluating the effect of Alzheimer’s disease-related biomarker change in corticobasal syndrome and progressive supranuclear palsy. Ann. Neurol..

[124] Urso D., Nigro S., Tafuri B., De Blasi R., Pereira J.B., Logroscino G. (2024). Nucleus basalis of Meynert degeneration predicts cognitive decline in corticobasal syndrome. Biol. Psychiatry.

[125] Schulz J., Pagano G., Fernández Bonfante J.A., Wilson H., Politis M. (2018). Nucleus basalis of Meynert degeneration precedes and predicts cognitive impairment in Parkinson’s disease. Brain.

[126] Ballarini T., Albrecht F., Mueller K., Jech R., Diehl-Schmid J., Fliessbach K., Kassubek J., Lauer M., Fassbender K., Schneider A. (2020). Disentangling brain functional network remodeling in corticobasal syndrome—A multimodal MRI study. Neuroimage Clin..

[127] Ju Y., Ou W., Su J., Averill C.L., Liu J., Wang M., Wang Z., Zhang Y., Liu B., Li L. (2020). White matter microstructural alterations in posttraumatic stress disorder: An ROI and whole-brain based meta-analysis. J. Affect. Disord..

[128] Kiehl K.A., Smith A.M., Mendrek A., Forster B.B., Hare R.D., Liddle P.F. (2004). Temporal lobe abnormalities in semantic processing by criminal psychopaths as revealed by functional magnetic resonance imaging. Psychiatry Res..

[129] Del Campo N., Phillips O., Ory-Magne F., Brefel-Courbon C., Galitzky M., Thalamas C., Narr K.L., Joshi S., Singh M.K., Péran P. (2021). Broad white matter impairment in multiple system atrophy. Hum. Brain Mapp..

[130] Pang H., Yu Z., Yu H., Li X., Bu S., Liu Y., Wang J., Zhao M., Fan G. (2024). Advanced cognitive patterns in multiple system atrophy compared to Parkinson’s disease: An individual diffusion tensor imaging study. Acad. Radiol..

[131] Kovacs G.G., Lukic M.J., Irwin D.J., Arzberger T., Respondek G., Lee E.B., Coughlin D., Giese A., Grossman M., Kurz C. (2020). Distribution patterns of tau pathology in progressive supranuclear palsy. Acta Neuropathol..

[132] Caso F., Agosta F., Volonté M.A., Ferraro P.M., Tiraboschi P., Copetti M., Valsasina P., Falautano M., Comi G., Falini A. (2016). Cognitive impairment in progressive supranuclear palsy-Richardson’s syndrome is related to white matter damage. Park. Relat. Disord..

[133] Adams N.E., Jafarian A., Perry A., Rouse M.A., Shaw A.D., Murley A.G., Cope T.E., Bevan-Jones W.R., Passamonti L., Street D. (2023). Neurophysiological consequences of synapse loss in progressive supranuclear palsy. Brain.

[134] Higuchi Y., Iwaki T., Tateishi J. (1995). Neurodegeneration in the limbic and paralimbic system in progressive supranuclear palsy. Neuropathol. Appl. Neurobiol..

[135] Ghosh B.C., Calder A.J., Peers P.V., Lawrence A.D., Acosta-Cabronero J., Pereira J.M., Hodges J.R., Rowe J.B. (2012). Social cognitive deficits and their neural correlates in progressive supranuclear palsy. Brain.

[136] Holland N., Savulich G., Jones P.S., Whiteside D.J., Street D., Swann P., Naessens M., Malpetti M., Hong Y.T., Fryer T.D. (2024). Differential synaptic loss in ß-amyloid positive versus ß-amyloid negative corticobasal syndrome. Mov. Disord..

[137] Tetreault A.M., Phan T., Petersen K.J., Claassen D.O., Neth B.J., Graff-Radford J., Albrecht F., Fliessbach K., Schneider A., Synofzik M. (2020). Network localization of alien limb in patients with corticobasal syndrome. Ann. Neurol..

[138] Constantinides V.C., Paraskevas G.P., Velonakis G., Stefanis L., Kapaki E. (2024). Localizing apraxia in corticobasal syndrome: A morphometric MRI study. Cereb. Cortex.

[139] Zhang L., Flagan T.M., Häkkinen S., Chu S.A., Brown J.A., Lee A.J., Pasquini L., Mandelli M.L., Gorno-Tempini M.L., Sturm V.E. (2023). Network connectivity alterations across the MAPT mutation clinical spectrum. Ann. Neurol..

[140] Yang H., Wang N., Luo X., Lv H., Liu H., Fan G. (2019). Altered functional connectivity of dentate nucleus in parkinsonian and cerebellar variants of multiple system atrophy. Brain Imaging Behav..

[141] Kawabata K., Hara K., Watanabe H., Bagarinao E., Ogura A., Masuda M., Yokoi T., Kato T., Ohdake R., Ito M. (2019). Alterations in cognition-related cerebello-cerebral networks in multiple system atrophy. Cerebellum.

[142] Li Y., Liu H., Yu H., Yang H., Guo M., Cao C., Pang H., Liu Y., Cao K., Fan G. (2023). Alterations of voxel-wise spontaneous activity and corresponding brain functional networks in multiple system atrophy patients with mild cognitive impairment. Hum. Brain Mapp..

[143] Yao Q., Zhu D., Li F., Xiao C., Lin X., Huang Q., Shi J. (2017). Altered functional and causal connectivity of cerebello-cortical circuits between multiple system atrophy (parkinsonian type) and Parkinson’s disease. Front. Aging Neurosci..

[144] Tang S., Wang Y., Liu Y., Chau S.W., Chan J.W., Chu W.C., Abrigo J.M., Mok V.C., Wing Y.K. (2022). Large-scale network dysfunction in alpha-synucleinopathy: A meta-analysis of resting-state functional connectivity. EBioMedicine.

[145] Ali F., Clark H., Machulda M., Senjem M.L., Lowe V.J., Jack C.R., Josephs K.A., Whitwell J., Botha H. (2024). Patterns of brain volume and metabolism predict clinical features in the progressive supranuclear palsy spectrum. Brain Commun..

[146] Gardner R.C., Boxer A.L., Trujillo A., Mirsky J.B., Guo C.C., Gennatas E.D., Heuer H.W., Fine E., Zhou J., Kramer J.H. (2013). Intrinsic connectivity network disruption in progressive supranuclear palsy. Ann. Neurol..

[147] Santangelo G., Cuoco S., Pellecchia M.T., Erro R., Barone P., Picillo M. (2018). Comparative cognitive and neuropsychiatric profiles between Parkinson’s disease, multiple system atrophy and progressive supranuclear palsy. J. Neurol..

[148] Warren N.M., Piggott M.A., Lees A.J., Burn D.J. (2007). The basal ganglia cholinergic neurochemistry of progressive supranuclear palsy and other neurodegenerative diseases. J. Neurol. Neurosurg. Psychiatry.

[149] Rau A., Jost W.H., Demerath T., Kellner E., Reisert M., Urbach H. (2022). Diffusion microstructure imaging in progressive supranuclear palsy: Reduced axonal volumes in the superior cerebellar peduncles, dentato-rubro-thalamic tracts, ventromedial thalami, and frontomesial white matter. Cereb. Cortex.

[150] Brown J.A., Hua A.Y., Trujllo A., Attygalle S., Binney R.J., Spina S., Lee S.E., Kramer J.H., Miller B.L., Rosen H.J. (2017). Advancing functional dysconnectivity and atrophy in progressive supranuclear palsy. Neuroimage Clin..

[151] Franzmeier N., Brendel M., Beyer L., Slemann L., Kovacs G.G., Arzberger T., Kurz C., Respondek G., Lukic M.J., Biel D. (2022). Tau deposition patterns are associated with functional connectivity in primary tauopathies. Nat. Commun..

[152] Abos A., Segura B., Baggio H.C., Campabadal A., Uribe C., Garrido A., Camara A., Muñoz E., Valldeoriola F., Marti M.J. (2019). Disrupted structural connectivity of fronto-deep gray matter pathways in progressive supranuclear palsy. Neuroimage Clin..

[153] Rosskopf J., Gorges M., Müller H.P., Lulé D., Uttner I., Ludolph A.C., Pinkhardt E., Juengling F.D., Kassubek J. (2017). Intrinsic functional connectivity alterations in progressive supranuclear palsy: Differential effects in frontal cortex, motor, and midbrain networks. Mov. Disord..

[154] Garraux G., Salmon E., Peigneux P., Kreisler A., Degueldre C., Lemaire C., Destée A., Franck G. (2000). Voxel-based distribution of metabolic impairment in corticobasal degeneration. Mov. Disord..

[155] Julayanont P., Burks D.W., Heilman K.M. (2019). Vertical and radial attentional neglect in corticobasal syndrome. Cogn. Behav. Neurol..

[156] Whiteside D.J., Street D., Murley A.G., Jones P.S., Malpetti M., Ghosh B.C.P., Coyle-Gilchrist I., Gerhard A., Hu M.T., Klein J.C. (2023). Network connectivity and structural correlates of survival in progressive supranuclear palsy and corticobasal syndrome. Hum. Brain Mapp..

[157] Mellema C.J., Nguyen K.P., Treacher A., Andrade A.X., Pouratian N., Sharma V.D., O’Suileabhain P., Montillo A.A. (2024). Longitudinal prognosis of Parkinson’s outcomes using causal connectivity. Neuroimage Clin..

[158] Keir G., Roytman M., Mashriqi F., Shahsavarani S., Franceschi A.M. (2024). Atypical parkinsonian syndromes: Structural, functional, and molecular imaging features. AJNR Am. J. Neuroradiol..

[159] Buchert R., Huppertz H.J., Wegner F., Berding G., Brendel M., Apostolova I., Buhmann C., Poetter-Nerger M., Dierks A., Katzdobler S. (2024). Added value of FDG-PET for detection of progressive supranuclear palsy. J. Neurol. Neurosurg. Psychiatry.

[160] Shen C., Chen L., Ge J.J., Lu J.Y., Chen Q.S., He S.J., Li X.Y., Zhao J., Sun Y.M., Wu P. (2021). Cerebral metabolism related to cognitive impairments in multiple system atrophy. Front. Neurol..

[161] Park K.W., Ko J.H., Choi N., Jo S., Park Y.J., Lee E.J., Kim S.J., Chung S.J., Lee C.S. (2020). Cortical hypometabolism associated with cognitive impairment of multiple system atrophy. Parkinsonism Relat. Disord..

[162] Shen C., Chen Q.S., Zuo C.T., Liu F.T., Wang J. (2022). The frontal and cerebellar metabolism related to cognitive dysfunction in multiple system atrophy. Front. Aging Neurosci..

[163] Hastings A., Cullinane P., Wrigley S., Revesz T., Morris H.R., Dickson J.C., Jaunmuktane Z., Warner T.T., De Pablo-Fernandez E. (2024). Neuropathologic validation and diagnostic accuracy of presynaptic dopaminergic imaging in the diagnosis of parkinsonism. Neurology.

[164] Bedard M.A., Aghourian M., Legault-Denis C., Postuma R.B., Soucy J.P., Gagnon J.F., Pelletier A., Montplaisir J. (2019). Brain cholinergic alterations in idiopathic REM sleep behaviour disorder: A PET imaging study with (18)F-FEOBV. Sleep Med..

[165] Niccolini F., Politis M. (2016). A systematic review of lessons learned from PET molecular imaging research in atypical parkinsonism. Eur. J. Nucl. Med. Mol. Imaging.

[166] Hirano S., Shinotoh H., Shimada H., Aotsuka A., Tanaka N., Ota T., Sato K., Ito H., Kuwabara S., Fukushi K. (2010). Cholinergic imaging in corticobasal syndrome, progressive supranuclear palsy and frontotemporal dementia. Brain.

[167] Black J.A., Pham N.T.T., Ali F., Machulda M.M., Lowe V.J., Josephs K.A., Whitwell J.L. (2024). Frontal hypometabolism in the diagnosis of progressive supranuclear palsy clinical variants. J. Neurol..

[168] Wang H., Wang B., Liao Y., Niu J., Chen M., Chen X., Dou X., Yu C., Zhong Y., Wang J. (2024). Identification of metabolic progression and subtypes in progressive supranuclear palsy by PET molecular imaging. Eur. J. Nucl. Med. Mol. Imaging.

[169] Roemer S.N., Brendel M., Gnörich J., Malpetti M., Zaganjori M., Quattrone A., Gross M., Steward A., Dewenter A., Wagner F. (2024). Subcortical tau is linked to hypoperfusion in connected cortical regions in 4-repeat tauopathies. Brain.

[170] Ye R., O’Callaghan C., Rua C., Hezemans F.H., Holland N., Malpetti M., Jones P.S., Barker R.A., Williams-Gray C.H., Robbins T.W. (2022). Locus coeruleus integrity from 7T MRI relates to apathy and cognition in parkinsonian disorders. Mov. Disord..

[171] Murley A.G., Rowe J.B. (2018). Neurotransmitter deficits from frontotemporal lobar degeneration. Brain.

[172] Ruberg M., Javoy-Agid F., Hirsch E., Scatton B., Lheureux R., Hauw J.J., Duyckaerts C., Gray F., Morel-Maroger A., Rascol A. (1985). Dopaminergic and cholinergic lesions in progressive supranuclear palsy. Ann. Neurol..

[173] Franceschi A.M., Clifton M., Naser-Tavakolian K., Ahmed O., Cruciata G., Bangiyev L., Clouston S., Franceschi D. (2020). ((18)F)-Fluorodeoxyglucose positron emission tomography/magnetic resonance imaging assessment of hypometabolism patterns in clinical phenotypes of suspected corticobasal degeneration. World J. Nucl. Med..

[174] Niethammer M., Tang C.C., Feigin A., Allen P.J., Heinen L., Hellwig S., Amtage F., Hanspal E., Vonsattel J.P., Poston K.L. (2014). A disease-specific metabolic brain network associated with corticobasal degeneration. Brain.

[175] Lutte I., Laterre C., Bodart J.M., De Volder A. (2000). Contribution of PET studies in diagnosis of corticobasal degeneration. Eur. Neurol..

[176] Pardini M., Huey E.D., Spina S., Kreisl W.C., Morbelli S., Wassermann E.M., Nobili F., Ghetti B., Grafman J. (2019). FDG-PET patterns associated with underlying pathology in corticobasal syndrome. Neurology.

[177] Isella V., Licciardo D., Ferri F., Crivellaro C., Morzenti S., Appollonio I.M., Ferrarese C. (2024). Left and right corticobasal syndrome: Comparison of cognitive profiles between metabolic imaging-matched groups. Neurol. Sci..

[178] Dodich A., Cerami C., Inguscio E., Iannaccone S., Magnani G., Marcone A., Guglielmo P., Vanoli G., Cappa S.F., Perani D. (2019). The clinico-metabolic correlates of language impairment in corticobasal syndrome and progressive supranuclear palsy. Neuroimage Clin..

[179] Madetko-Alster N., Otto-Slusarczyk D., Struga M., Kutylowski M., Drzewinska A., Duszynska-Was K., Migda B., Alster P. (2024). Glucose metabolism and cognitive decline in progressive supranuclear palsy and corticobasal syndrome: A preliminary study. J. Clin. Med..

[180] Erlinger M., Molina-Ruiz R., Brumby A., Cordas D., Hunter M., Ferreiro Arguelles C., Yus M., Owens-Walton C., Jakabek D., Shaw M. (2023). Striatal and thalamic automatic segmentation, morphology, and clinical correlates in Parkinsonism: Parkinson’s disease, multiple system atrophy and progressive supranuclear palsy. Psychiatry Res. Neuroimaging.

[181] Seki M., Seppi K., Mueller C., Potrusil T., Goebel G., Reiter E., Nocker M., Kremser C., Wildauer M., Schocke M. (2019). Diagnostic potential of multimodal MRI markers in atypical parkinsonian disorders. J. Parkinsons Dis..

[182] Shao N., Yang J., Li J., Shang H.F. (2014). Voxelwise meta-analysis of gray matter anomalies in progressive supranuclear palsy and Parkinson’s disease using anatomic likelihood estimation. Front. Hum. Neurosci..

[183] Wang N., Zhang L., Yang H., Luo X., Fan G. (2019). Do multiple system atrophy and Parkinson’s disease show distinct patterns of volumetric alterations across hippocampal subfields? An exploratory study. Eur. Radiol..

[184] Chunowski P., Madetko-Alster N., Alster P. (2024). symmetry in atypical parkinsonian syndromes—A review. J. Clin. Med..

[185] Hess C.P., Christine C.W., Apple A.C., Dillon W.P., Aminoff M.J. (2014). Changes in the thalamus in atypical parkinsonism detected using shape analysis and diffusion tensor imaging. AJNR Am. J. Neuroradiol..

[186] Erbetta A., Mandelli M.L., Savoiardo M., Grisoli M., Bizzi A., Soliveri P., Chiapparini L., Prioni S., Bruzzone M.G., Girotti F. (2009). Diffusion tensor imaging shows different topographic involvement of the thalamus in progressive supranuclear palsy and corticobasal degeneration. AJNR Am. J. Neuroradiol..

[187] Chougar L., Arsovic E., Gaurav R., Biondetti E., Faucher A., ValabrÃ¨gue R., Pyatigorskaya N., Dupont G., Lejeune F.X., Cormier F. (2022). Regional selectivity of neuromelanin changes in the substantia nigra in atypical parkinsonism. Mov. Disord..

[188] Nobileau A., Gaurav R., Chougar L., Faucher A., Valabrègue R., Mangone G., Leu-Semenescu S., Lejeune F.X., Corvol J.C., Arnulf I. (2023). A neuromelanin-sensitive magnetic resonance imaging changes in the locus coeruleus/subcoeruleus complex in patients with typical and atypical parkinsonism. Mov. Disord..

[189] Shah A., Prasad S., Indoria A., Pal P.K., Saini J., Ingalhalikar M. (2024). Free water imaging in Parkinson’s disease and atypical parkinsonian disorders. J. Neurol..

[190] Mohammadi S., Ghaderi S. (2024). Parkinson’s disease and Parkinsonism syndromes: Evaluating iron deposition in the putamen using magnetic susceptibility MRI techniques—A systematic review and literature analysis. Heliyon.

[191] Kim M.S., Park D.G., Shin I.J., An Y.S., Yoon J.H. (2024). The role of dual-phase 18 F-FP-CIT PET to early diagnosis of corticobasal syndrome. Clin. Nucl. Med..

[192] Bharti K., Bologna M., Upadhyay N., Piattella M.C., Suppa A., Petsas N., Giannì C., Tona F., Berardelli A., Pantano P. (2017). Abnormal resting-state functional connectivity in progressive supranuclear palsy and corticobasal syndrome. Front. Neurol..

[193] Parmera J.B., de Godoi Carneiro C., de Almeida I.J., de Oliveira M.C.B., Barbosa P.M., Studart-Neto A., Ono C.R., Nitrini R., Buchpiguel C.A., Barbosa E.R. (2024). Probable 4-repeat tauopathy criteria predict brain amyloid negativity, distinct clinical features, and FDG-PET/MRI neurodegeneneration patterns in corticobasal syndrome. Mov. Disord. Clin. Pract..

[194] Mantovani E., Martini A., Dinoto A., Zucchella C., Ferrari S., Mariotto S., Tinazzi M., Tamburin S. (2024). Biomarkers for cognitive impairment in alpha-synucleinopathies: An overview of systematic reviews and meta-analyses. NPJ Park. Dis..

[195] Katzdobler S., Nübling G., Klietz M., Fietzek U.M., Palleis C., Bernhardt A.M., Wegner F., Huber M., Rogozinski S., Schneider L.S. (2024). GFAP and NfL as fluid biomarkers for clinical disease severity and disease progression in multiple system atrophy (MSA). J. Neurol..

[196] Ashton N.J., Janelidze S., Al Khleifat A., Leuzy A., van der Ende E.L., Karikari T.K., Benedet A.L., Pascoal T.A., Lleó A., Parnetti L. (2021). A multicentre validation study of the diagnostic value of plasma neurofilament light. Nat. Commun..

[197] Lenska-Mieciek M., Madetko-Alster N., Alster P., Królicki L., Fiszer U., Koziorowski D. (2023). Inflammation in multiple system atrophy. Front. Immunol..

[198] Hall S., Janelidze S., Surova Y., Widner H., Zetterberg H., Hansson O. (2018). Cerebrospinal fluid concentrations of inflammatory markers in Parkinson’s disease and atypical parkinsonian disorders. Sci. Rep..

[199] Ryman S.G., Poston K.L. (2020). MRI biomarkers of motor and non-motor symptoms in Parkinson’s disease. Park. Relat. Disord..

[200] Zhang L., Li R., Hou Y., Cao B., Wei Q., Ou R., Liu K., Lin J., Yang T., Xiao Y. (2022). Cystatin C predicts cognitive decline in multiple system atrophy: A 1-year prospective cohort study. Front. Aging Neurosci..

[201] Fernandes Gomes B., Kumar A., Ashton N.J., Hall S., Stomrud E., Smith R., Zetterberg H., Blennow K., Mattsson-Carlgren N., Hansson O. (2024). Corticotropin-releasing hormone as a candidate biomarker for parkinsonian disorders. Brain Commun..

[202] Pereira J.B., Kumar A., Hall S., Palmqvist S., Stomrud E., Bali D., Parchi P., Mattsson-Carlgren N., Janelidze S., Hansson O. (2023). DOPA decarboxylase is an emerging biomarker for Parkinsonian disorders including preclinical Lewy body disease. Nat. Aging.

[203] Mancini A., Stoops E., Demeyer L., Bellomo G., Paolini Paoletti F., Gaetani L., Di Filippo M., Parnetti L. (2023). LRRK2 quantification in cerebrospinal fluid of patients with Parkinson’s disease and atypical parkinsonian syndromes. Mov. Disord..

[204] Palleis C., Franzmeier N., Weidinger E., Bernhardt A.M., Katzdobler S., Wall S., Ferschmann C., Harris S., Schmitt J., Schuster S. (2024). Association of neurofilament light chain, [(18)F]PI-2620 tau-PET, TSPO-PET, and clinical progression in patients with ß-amyloid-negative CBS. Neurology.

[205] Wu L., Gilyazova N., Ervin J.F., Wang S.J., Xu B. (2022). Site-specific phospho-tau aggregation-based biomarker discovery for ad diagnosis and differentiation. ACS Chem. Neurosci..

[206] Giagkou N., Kapsali I., Brinia M.E., Constantinides V.C. (2024). Cerebrospinal fluid total and phosphorylated tau protein in behavioral variant frontotemporal dementia, progressive supranuclear palsy, corticobasal syndrome and non-fluent agrammatic primary progressive aphasia: A systematic review and meta-analysis. Biomedicines.

[207] Nilsson J., Constantinescu J., Nellgård B., Jakobsson P., Brum W.S., Gobom J., Forsgren L., Dalla K., Constantinescu R., Zetterberg H. (2023). Cerebrospinal fluid biomarkers of synaptic dysfunction are altered in Parkinson’s disease and related disorders. Mov. Disord..

[208] Vacchi E., Lazzarini E., Pinton S., Chiaro G., Disanto G., Marchi F., Robert T., Staedler C., Galati S., Gobbi C. (2022). Tau protein quantification in skin biopsies differentiates tauopathies from alpha-synucleinopathies. Brain.

[209] Li Q., Li Z., Han X., Shen X., Wang F., Bai L., Zhang R., Wang Y., Zhu X. (2022). A panel of plasma biomarkers for differential diagnosis of parkinsonian syndromes. Front. Neurosci..

[210] Huang S.Y., Chen S.F., Cui M., Zhao M., Shen X.N., Guo Y., Zhang Y.R., Zhang W., Wang H.F., Huang Y.Y. (2023). Plasma biomarkers and positron emission tomography tau pathology in progressive supranuclear palsy. Mov. Disord..

[211] Chen Y., Huang J., Li Y., Chen X., Ye Q. (2025). Diagnostic value of six plasma biomarkers in progressive supranuclear palsy, multiple system atrophy, and Parkinson’s disease. Clin. Chim. Acta.

[212] Farrell K., Humphrey J., Chang T., Zhao Y., Leung Y.Y., Kuksa P.P., Patil V., Lee W.P., Kuzma A.B., Valladares O. (2024). Genetic, transcriptomic, histological, and biochemical analysis of progressive supranuclear palsy implicates glial activation and novel risk genes. Nat. Commun..

[213] Wang H., Chang T.S., Dombroski B.A., Cheng P.L., Patil V., Valiente-Banuet L., Farrell K., McLean C., Molina-Porcel L., Rajput A. (2024). Whole-genome sequencing analysis reveals new susceptibility loci and structural variants associated with progressive supranuclear palsy. Mol. Neurodegener..

[214] Langerscheidt F., Wied T., Al Kabbani M.A., van Eimeren T., Wunderlich G., Zempel H. (2024). Genetic forms of tauopathies: Inherited causes and implications of Alzheimer’s disease-like TAU pathology in primary and secondary tauopathies. J. Neurol..

[215] Stefanova N., Wenning G.K. (2023). Multiple system atrophy: At the crossroads of cellular, molecular and genetic mechanisms. Nat. Rev. Neurosci..

[216] Koga S., Josephs K.A., Aiba I., Yoshida M., Dickson D.W. (2022). Neuropathology and emerging biomarkers in corticobasal syndrome. J. Neurol. Neurosurg. Psychiatry.

[217] Montalbano M., Majmundar L., Sengupta U., Fung L., Kayed R. (2023). Pathological tau signatures and nuclear alterations in neurons, astrocytes and microglia in Alzheimer’s disease, progressive supranuclear palsy, and dementia with Lewy bodies. Brain Pathol..

[218] Kaindlstorfer C., García J., Winkler C., Wenning G.K., Nikkhah G., Döbrössy M.D. (2012). Behavioral and histological analysis of a partial double-lesion model of parkinson-variant multiple system atrophy. J. Neurosci. Res..

[219] Lai T.T., Gericke B., Feja M., Conoscenti M., Zelikowsky M., Richter F. (2023). Anxiety in synucleinopathies: Neuronal circuitry, underlying pathomechanisms and current therapeutic strategies. NPJ Park. Dis..

[220] Gregersen E., Betzer C., Kim W.S., Kovacs G., Reimer L., Halliday G.M., Thiel S., Jensen P.H. (2021). Alpha-synuclein activates the classical complement pathway and mediates complement-dependent cell toxicity. J. Neuroinflamm..

[221] Jucaite A., Cselényi Z., Kreisl W.C., Rabiner E.A., Varrone A., Carson R.E., Rinne J.O., Savage A., Schou M., Johnström P. (2022). Glia imaging differentiates multiple system atrophy from Parkinson’s disease: A positron emission tomography study with [(11) C]PBR28 and machine learning analysis. Mov. Disord..

[222] Koga S., Parks A., Uitti R.J., van Gerpen J.A., Cheshire W.P., Wszolek Z.K., Dickson D.W. (2017). Profile of cognitive impairment and underlying pathology in multiple system atrophy. Mov. Disord..

[223] Ando T., Riku Y., Akagi A., Miyahara H., Hirano M., Ikeda T., Yabata H., Koizumi R., Oba C., Morozumi S. (2022). Multiple system atrophy variant with severe hippocampal pathology. Brain Pathol..

[224] Jellinger K.A. (2022). Heterogeneity of multiple system atrophy: An update. Biomedicines.

[225] Van der Perren A., Gelders G., Fenyi A., Bousset L., Brito F., Peelaerts W., Van den Haute C., Gentleman S., Melki R., Baekelandt V. (2020). The structural differences between patient-derived alpha-synuclein strains dictate characteristics of Parkinson’s disease, multiple system atrophy and dementia with Lewy bodies. Acta Neuropathol..

[226] Böing C., Di Fabrizio M., Burger D., Bol J.G.J.M., Huisman E., Rozemuller A.J.M., van de Berg W.D.J., Stahlberg H., Lewis A.J. (2024). Distinct ultrastructural phenotypes of glial and neuronal alpha-synuclein inclusions in multiple system atrophy. Brain.

[227] Briel N., Pratsch K., Roeber S., Arzberger T., Herms J. (2021). Contribution of the astrocytic tau pathology to synapse loss in progressive supranuclear palsy and corticobasal degeneration. Brain Pathol..

[228] Backman E., Gardberg M., Luntamo L., Peurla M., Vahlberg T., Borghammer P., Stefanova N., Wenning G., Kaasinen V. (2024). Nigral Neuroinflammation and Dopaminergic Neurons in PD, MSA and PSP: A Comparative Clinicopathological Study (Abstract). Mov. Disord..

[229] Malpetti M., Roemer S.N., Harris S., Gross M., Gnörich J., Stephens A., Dewenter A., Steward A., Biel D., Dehsarvi A. (2024). Neuroinflammation parallels 18F-PI-2620 positron emission tomography patterns in primary 4-repeat tauopathies. Mov. Disord..

[230] Ferschmann C., Messerschmidt K., Gnörich J., Barthel H., Marek K., Palleis C., Katzdobler S., Stockbauer A., Fietzek U., Finze A. (2024). Tau accumulation is associated with dopamine deficiency in vivo in four-repeat tauopathies. Eur. J. Nucl. Med. Mol. Imaging.

[231] Ling H., Kovacs G.G., Vonsattel J.P., Davey K., Mok K.Y., Hardy J., Morris H.R., Warner T.T., Holton J.L., Revesz T. (2016). Astrogliopathy predominates the earliest stage of corticobasal degeneration pathology. Brain.

[232] Yan X., Uronen R.L., Huttunen H.J. (2020). The interaction of alpha-synuclein and tau: A molecular conspiracy in neurodegeneration?. Semin. Cell Dev. Biol..

[233] Ramirez J., Saleh I.G., Yanagawa E.S.K., Shimogawa M., Brackhahn E., Petersson E.J., Rhoades E. (2024). Multivalency drives interactions of alpha-synuclein fibrils with tau. PLoS ONE.

[234] Jin M., Wang S., Gao X., Zou Z., Hirotsune S., Sun L. (2024). Pathological and physiological functional cross-talks of Î±-synuclein and tau in the central nervous system. Neural Regen. Res..

[235] Didonna A. (2020). Tau at the interface between neurodegeneration and neuroinflammation. Genes. Immun..

[236] Bartolome F., Carro E., Alquezar C. (2022). Oxidative stress in tauopathies: From cause to therapy. Antioxidants.

[237] Palleis C., Sauerbeck J., Beyer L., Harris S., Schmitt J., Morenas-Rodriguez E., Finze A., Nitschmann A., Ruch-Rubinstein F., Eckenweber F. (2021). In vivo assessment of neuroinflammation in 4-repeat tauopathies. Mov. Disord..

[238] Weintraub D., Aarsland D., Biundo R., Dobkin R., Goldman J., Lewis S. (2022). Management of psychiatric and cognitive complications in Parkinson’s disease. BMJ.

[239] Moretti D.V., Binetti G., Zanetti O., Frisoni G.B. (2014). Non-ergot dopamine agonist rotigotine as a promising therapeutic tool in atypical parkinsonism syndromes: A 24 months pilot observational open-label study. Neuropharmacology.

[240] Forlenza O.V., Aprahamian I., de Paula V.J., Hajek T. (2016). Lithium, a therapy for AD: Current evidence from clinical trials of neurodegenerative disorders. Curr. Alzheimer Res..

[241] Novak P., Pimentel Maldonado D.A., Novak V. (2019). Safety and preliminary efficacy of intranasal insulin for cognitive impairment in Parkinson disease and multiple system atrophy: A double-blinded placebo-controlled pilot study. PLoS ONE.

[242] Zhang M., He T., Wang Q. (2021). Effects of non-invasive brain stimulation on multiple system atrophy: A systematic review. Front. Neurosci..

[243] Lozupone M., Dibello V., Daniele A., Solfrizzi V., Resta E., Panza F. (2024). How can we manage progressive supranuclear palsy syndrome with pharmacotherapy?. Expert. Opin. Pharmacother..

[244] Panza F., Dibello V., Sardone R., Castellana F., Zupo R., Lampignano L., Bortone I., Stallone R., Cirillo N., Damiani C. (2023). Clinical development of passive tau-based immunotherapeutics for treating primary and secondary tauopathies. Expert. Opin. Investig. Drugs.

[245] Ma J., Zhang G., Zhao Z., Chan P., Ye Z. (2024). Levodopa modulates semantic fluency and uniqueness in non-demented patients with progressive supranuclear palsy. Brain Behav..

[246] Metta V., Chaudhuri K.R., Leta V., Mridula K.R., Koduri C., Deepak S., Kalpala R., Reddy N., Chung-Faye G., Borgohain R. (2022). Real-life benefits of intrajejunal levodopa infusion therapy in four patients with the parkinsonian variant of progressive supranuclear palsy: A 1-year follow-up data report. Brain Behav..

[247] Rittman T., Coyle-Gilchrist I.T., Rowe J.B. (2016). Managing cognition in progressive supranuclear palsy. Neurodegener. Dis. Manag..

[248] Alexoudi A., Patrikelis P., Deftereos S., Fasilis T., Karakalos D., Verentzioti A., Korfias S., Sakas D., Gatzonis S. (2019). Effects of anodal transcranial direct current stimulation on cognitive dysfunction in patients with progressive supranuclear palsy. Psychiatriki.

[249] Mimura Y., Tobari Y., Nakahara K., Nakajima S., Yoshida K., Mimura M., Noda Y. (2023). Transcranial magnetic stimulation neurophysiology in patients with non-Alzheimer’s neurodegenerative diseases: A systematic review and meta-analysis. Neurosci. Biobehav. Rev..

[250] Bluett B., Pantelyat A.Y., Litvan I., Ali F., Apetauerova D., Bega D., Bloom L., Bower J., Boxer A.L., Dale M.L. (2021). Best practices in the clinical management of progressive supranuclear palsy and corticobasal syndrome: A consensus statement of the curepsp centers of care. Front. Neurol..

[251] Romero J.P., Martínez-Benito A., de Noreña D., Hurtado-Martínez A., Sánchez-Cuesta F.J., González-Zamorano Y., Moreno-Verdú M. (2024). Combined non-invasive neuromodulation using transcranial direct current stimulation, motor imagery and action observation for motor, cognitive and functional recovery in cortico-basal degeneration: A single case study. EXCLI J..

